# Novel enhancement of stability and antimicrobial activity of beetroot pigment nanocomposites via graphene oxide and silver nanoparticles

**DOI:** 10.1038/s41598-026-42211-w

**Published:** 2026-03-26

**Authors:** Habiba A. Ahmed, Abeer E. Abd El-Wahab, Sara Gad

**Affiliations:** 1 Plant Biochemistry Department, National Research Centre, Dokki, 12622 Giza Egypt; 2 Medical Biotechnology Department, Institute of Genetic Engineering and Biotechnology, City of Scientific Research and Technological Applications (SRTA-City), New Borg EL-Arab, Alexandria, 21934 Egypt; 3 Pharmaceutical and Fermentation Industries Development Center, City of Scientific Research and Technological Applications (SRTA-City), New Borg EL-Arab, Alexandria, 21934 Egypt; 4 Electronic Materials Research Department, Advanced Technology and New Materials Research Institute, City of Scientifc Research and Technological Applications (SRTA-City), Alexandria, Egypt

**Keywords:** Beta vulgaris pigments, Graphene oxide (GO), Silver (Ag) nanoparticles, Antimicrobial activity, Biochemistry, Biological techniques, Biotechnology, Chemistry, Materials science, Microbiology, Nanoscience and technology

## Abstract

Natural pigments like betalains have strong antimicrobial properties but degrade quickly under environmental stress, limiting their stability in practical applications. This study presents the green synthesis of silver (Ag) and graphene oxide (GO) nanomaterials using a chitosan assisted method and their incorporation into beta vulgaris pigments to enhance overall performance. Trace amounts of pre-synthesized silver nanoparticles and graphene oxide were added to beta vulgaris ethanolic extract and subsequently embedded within a xanthan-gum polymer matrix to enhance pigments stability in beta vulgaris extract. This bioactive formulation improves the stability of sensitive pigments including anthocyanins and betacyanins by reducing degradation, while also strengthening antimicrobial activity due to the intrinsic properties of Ag and GO. Synthesized metals Ag, GO and different mixture of beta vulgaris extract with metals (pigment-Ag, pigment-Go, pigment-Ag-Go, pigment with xanthan gum and free xanthan gum) were characterized with the UV-Vis spectroscopy, fluorescence spectroscopy FTIR, Raman, SEM and TEM. Antimicrobial activity was evaluated against Gram-negative (*Escherichia coli*, *Salmonella typhi*), Gram-positive (*Staphylococcus aureus*,* Streptococcus mutans*), and fungal (*Candida albicans*) strains using both Agar well diffusion and microplate MIC assays. Inhibition zones increased with concentration (25, 50, and 100 mg/mL), with Pig-Ag-GO showing the highest activity at 100 mg/mL, producing zones up to 29 mm, especially against *S. aureus*. MICs were determined using concentrations ranging from 50 to 1000 µg/mL. The control pigment showed moderate activity, with MICs ranging from 247 to 333 µg/mL for Gram-negative bacteria, 108 µg/mL for *S. aureus*, and 717 µg/mL for *C. albicans*. Pig-Ag and Pig-GO exhibited improved efficacy, while the Pig-Ag-GO nanocomposite demonstrated the lowest MICs across all strains 200 µg/mL for *E. coli*, 217 µg/mL for *S. typhi*, 68 µg/mL for *S. aureus*, and 600 µg/mL for *C. albicans*. These results confirm the synergistic antimicrobial effect of combining silver and graphene oxide with natural pigment, enhancing both activity and stability. This study highlights a sustainable alternative to conventional chemical methods, utilizing green synthesis for metal nanoparticles to enhance pigment stability and maintain long-term antimicrobial properties.

**Supplementary Information:**

The online version contains supplementary material available at 10.1038/s41598-026-42211-w.

## Introduction

Nanotechnology offers powerful tools for developing next-generation antimicrobial agents, especially as antimicrobial resistance (AMR) continues to rise and is recognized by the World Health Organization as a major global health threat. Nanocomposites combining natural biomolecules with functional nanomaterials have gained attention due to their strong microbial inhibition, oxidative stress induction, membrane disruption, and reduced likelihood of resistance development. Among natural bioactive, beetroot (Beta vulgaris) pigments rich in betalains exhibit antioxidant, anti-inflammatory, and antimicrobial properties; however, their rapid degradation under light, heat, oxygen, and pH fluctuations limits practical use. To improve pigment stability, natural polymers are increasingly used^1–3^. Xanthan gum, an anionic heteropolysaccharide, provides excellent viscosity, biocompatibility, and film-forming ability, creating a protective network that reduces oxidation and photodegradation while sterically stabilizing dispersed nanoparticles^4,5^. These characteristics make xanthan gum an effective encapsulant for pigment nanomaterial hybrids. Incorporating betalains into nanomaterials not only enhances their stability but also improves their bioactivity, making them more effective for biomedical applications. Silver nanoparticles (Ag-NPs) are among the most potent antimicrobial nanomaterials, capable of generating reactive oxygen species (ROS) that damage microbial membranes, proteins, and DNA^6,7^. Green-synthesized Ag-NPs, produced using chitosan or plant extracts rich in natural phytochemicals, provide an eco-friendly and sustainable approach to nanoparticle fabrication. These biosynthesized nanoparticles exhibit strong antibacterial activity against both Gram-positive and Gram-negative bacteria, as well as notable anticancer effects, highlighting their dual therapeutic potential^8–10^. The synergistic combination of natural pigments and nanomaterials thus offers enhanced stability, bioactivity, and multifunctionality, making them promising candidates for future biomedical and health-related applications. while graphene oxide (GO) exhibits high surface area, abundant oxygen-containing groups, and intrinsic antimicrobial activity that disrupt microbial membranes. Their combination (Ag-GO) produces strong synergistic antimicrobial effects and improved dispersion due to GO’s functional groups. Green synthesis approaches have gained importance as sustainable alternatives to conventional chemical routes, with chitosan frequently serving as a biocompatible reducing and stabilizing agent for metal nanoparticles^11^. The proposed Ag-GO-betalain-xanthan gum system advances existing literature by integrating dual functionality: enhanced antimicrobial activity and improved pigment stability, which has not been addressed in previous Ag and GO studies that focus primarily on antimicrobial applications^12–14^. Unlike conventional Ag and GO nanocomposites, our formulation incorporates natural betalain pigments from *Beta vulgaris*, providing additional bioactive properties, and employs xanthan gum as a stabilizing polymer to prevent nanoparticle agglomeration and reduce pigment degradation^15^. This combination creates a synergistic interaction among the metal nanomaterials, natural pigments, and polymer matrix, leading to functional and structural improvements over earlier Ag/GO systems^16^. This eco-friendly nanocomposite design offers improved durability and antimicrobial efficacy while supporting sustainable development goals through reduced chemical use and enhanced health-related applications^17^. Through this approach, the study contributes to SDG 3 (Good Health and Well-being) by addressing antimicrobial resistance, and SDG 12 (Responsible Consumption and Production) by promoting the use of sustainable materials in nanotechnology development.

## Materials and methods

All chemicals utilized in this study were of analytical grade and purchased from sigma Aldrich (USA). microbial strains were sourced from the American type of culture collection (ATCC) in Manassas, VA, USA, ensuring standardized and reliable strains for the study.

### Preparation of silver nanoparticles

The synthesis of chitosan-coated silver nanoparticles (Ag NPs) was carried out following an environmentally friendly approach. A 20 mL solution of chitosan (99.9%, Sigma-Aldrich) was prepared in 1% (v/v) acetic acid, to which 2 mL of 10⁻² M silver nitrate (AgNO₃, 99.9%, Sigma-Aldrich) was added. The mixture was stirred until homogeneous and then transferred to an amber bottle, which was incubated at 95 °C for 12 h. During this process, the solution changed color from colorless to bright yellow and then to yellowish-brown, indicating the reduction of Ag⁺ ions to silver nanoparticles. Finally, the reaction product was lyophilized to remove any residual solvent, then dried at 50 °C for 24 h (Ag NPs)^18^.

### Preparation of graphene oxide

As reported in several previous studies, GO nanosheets were synthesized by the oxidation of natural graphite using a modified Hummers method (Salah et al., 2023)^19^. Potassium permanganate (3 g) was gradually added to a mixture of graphite powder (0.5 g), sulfuric acid (25 mL, 96%), and sodium nitrate (0.5 g) at a temperature of approximately 0 °C. The resulting mixture was then placed in a water bath and maintained at 35 °C for 1 h. Subsequently, 50 mL of deionized water was added, followed by the addition of 5 mL hydrogen peroxide (30%), with continuous stirring. The precipitate was then separated from the solution by high-speed centrifugation. The resulting graphene oxide (GO) was subjected to several washing cycles and dried at approximately 50 °C. Finally, the dispersion of the synthesized GO was achieved by sonicating it in deionized water for about 1 h.

### Preparation of silver and graphene oxide

Various methods have been reported for the synthesis of GO/Ag nanocomposites (NC), including chemical reduction, thermal reduction, and photochemical reduction. In these processes, GO suspension in water or ethanol interacts with a silver precursor in the presence of a reducing agent such as sodium citrate or polymers, facilitating the reduction of metal ions to form GO/Ag NCs. In this study, a novel and highly stable GO/Ag nanocomposite was synthesized via an ex-situ method by gradually adding 8 mL of silver nanoparticles (Ag NPs) to 8 mL of graphene oxide (GO) colloid in a glass beaker over the course of 2 h, maintaining a 1:1 volume ratio between GO and Ag NPs. The mixture was then homogenized for 45 min under sonication at room temperature. The resulting Ag/GO NPs were subsequently dried using a lyophilizer^20^.

### Extract and stabilize red pigments of Beta vulgaris using metals nanoparticles and Xanthan gum

**Fig. 1:**
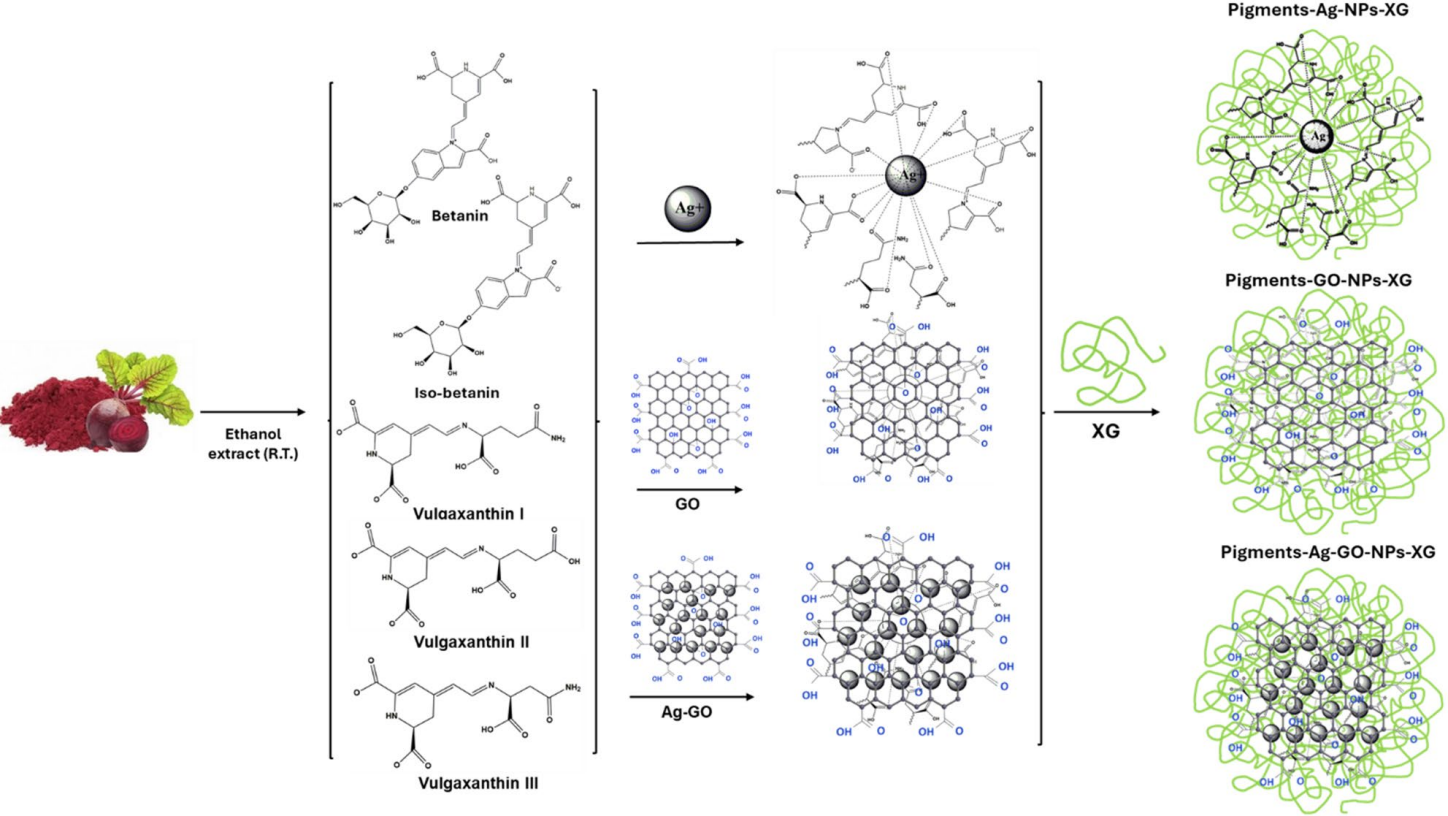
The scheme of the preparation of xanthan gum (XG) stabilized pigments of beta vulgaris using Graphene oxide (GO) and silver (Ag) nanoparticles.

To extract and stabilize red pigments from *Beta vulgaris*, 10 g of beetroot powder were soaked in 200 mL of 70% ethanol acidified with citric acid. The mixture was homogenized for 1 h, stirred for 2 h, and then filtered using Whatman No. 1 filter paper. The solvent was evaporated using a rotary evaporator, yielding 40 mL of crude extract at 100 mg/mL. To enhance stability, 0.05 g of xanthan gum were added as a polymer and stirred for 1 h. Each 10 mL portion of the mixture was then treated separately with 500 µL of silver nanoparticles (Ag-NPs), graphene oxide nanoparticles (GO-NPs), or (Ag/GO NPs), while a control sample remained untreated. Finally, the mixtures were sonicated for 15 min to ensure uniform dispersion (Fig. 1)^22^.

**Physical characterization**: The physical characterization of the synthesized nanomaterials was conducted using a range of advanced analytical techniques. Fourier-transform infrared spectroscopy (FTIR, Shimadzu 7000, resolution: 4 cm^−1^) was employed to identify functional groups and confirm bonding structures. Raman spectroscopy (Sentira *inVia*, with 532 nm laser excitation) was used to analyze vibrational modes and assess phase purity. The crystalline structure of the Ag nanoparticles and graphene oxide nanomaterials was examined by X-ray diffraction (XRD). Measurements were performed using Cu Kα radiation, and scans were collected over an appropriate 2θ range. The obtained diffraction patterns were analyzed to identify the crystalline phases present and to evaluate changes in structural order after synthesis. Surface morphology and particle size distribution were examined using scanning electron microscopy (SEM, JEOL JSM-7600 F, operated at 5–15 kV), while internal structural features and crystallite boundaries were observed via transmission electron microscopy (TEM, JEOL, operated at 120 kV). Elemental composition and distribution were determined through EDX analysis integrated into the TEM system. Optical properties were evaluated using UV-Vis spectroscopy (Shimadzu UV-2600, scanning from 200 to 800 nm), and fluorescence spectroscopy (Bruker, equipped with a xenon lamp), which provided insights into band gaps and emission characteristics relevant to sensing applications^22^.

**Stability of pigments**: To evaluate the stability of the pigments, 1 mL of the sample was uniformly spread as a thin film onto a clean glass slide. The slide was observed under both ambient light and UV illumination for any visible color changes. Observations were recorded immediately after application and periodically over a three-month period to monitor stability over time. Photographs were taken at specific intervals: after 1 day, 3 days, 1 week, 3 weeks, 6 weeks, and 3 months.

### Antimicrobial activity

The well diffusion technique was used to evaluate the antimicrobial activity of different formulations, including the control (pigment with Xanthan gum), Xanthan gum alone, control with silver (Ag-NPs), control with graphene oxide (GO-NPs), and control with Ag/GO-NPs. Antimicrobial activity was tested against various Gram-positive bacteria, including *S. aureus* (ATCC 13565) Streptococcus mutans (ATCC 25175) and Gram-negative bacteria, including *E. coli* (ATCC 25922) and *S. typhimurium* (ATCC 14028). The antifungal activity was examined against one fungal species (*C. albicans* ATCC 10231). The antibacterial activity was assessed using nutrient agar medium, with incubation at 37 °C for 24 h, followed by measurement of inhibition zones in millimeters (mm) using a ruler. The antifungal activity was evaluated on potato dextrose agar (PDA), with inhibition zones measured after 48 h of incubation at 25°C^23^.

**Microplate Reader Assay for MIC (Minimum Inhibitory Concentration) Evaluation**: The antibacterial efficacy of the samples was assessed using a 96-well microplate-based assay, adapted from the protocol described by Bechert et al. (2000)^24^, with minor modifications. Each well was inoculated with 100 µL of bacterial suspension (~ 10⁶ CFU/µL) prepared in LB broth. Subsequently, 100 µL of the test formulations comprising the control (natural pigment with Xanthan gum), Xanthan gum alone, pigment combined with silver nanoparticles (Pig-Ag), with graphene oxide (Pig-GO), and with both silver and graphene oxide (Pig-Ag-GO) were added to the wells in triplicate. The plates were incubated at 37 °C for 24 h under micro-aerophilic conditions. Post incubation and bacterial growth was quantified by measuring absorbance at 620 nm using an ELISA microplate reader. Antibacterial activity was expressed as the percentage inhibition of bacterial growth, calculated using a standard formula.

$${\rm Inhibition} (\%) = (A-A_{1})/(A_{10}-A_{1}) \times 100$$

where A is the absorbance of the treatment group, A₀ is the absorbance of the control group (bacteria without treatment), and A₁ is the absorbance of the blank (media without bacteria).

### Statistical analysis

All data are presented as the mean ± standard deviation (SD) from three independent experiments, each conducted in triplicate. Statistical analyses were carried out using CoStat for Windows (version 6.45), and differences were considered significant at *p* < 0.05.

## Results and discussions

### Physical characterization

#### Fourier transform infrared spectroscopy (FTIR)

**Fig. 2:**
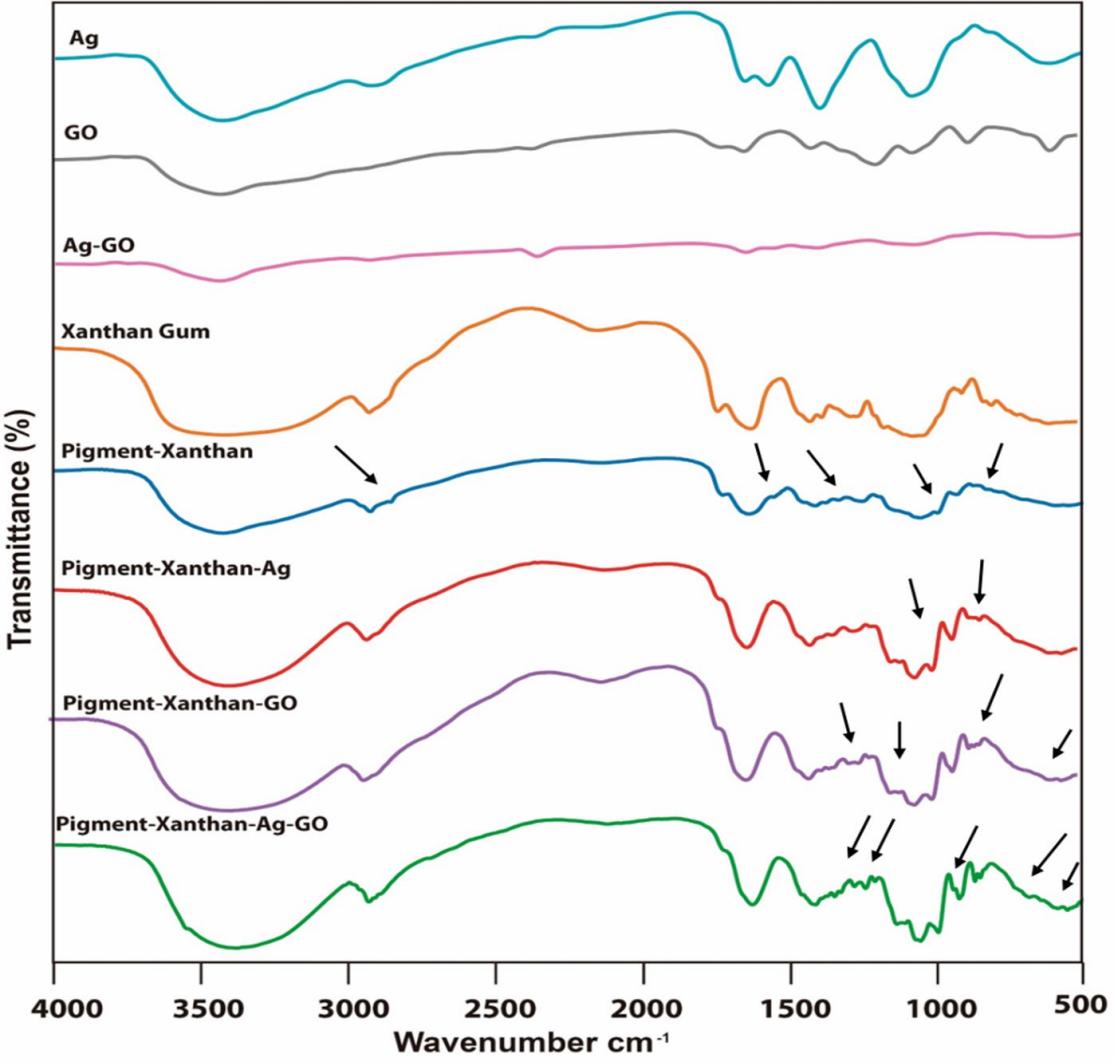
FTIR spectra of Ag nanoparticles, graphene oxide (GO), silver-graphene oxide (Ag/GO), xanthan gum, pigment-xanthan, pigment-xanthan-Ag, pigment-xanthan-GO, and pigment-xanthan-Ag/GO. Black arrows indicate the shifted peaks and newly appeared bands resulting from interaction between the components.

Figure 2 shows the FTIR spectra of silver nanoparticles (Ag-NPs), graphene oxide (GO), silver- graphene oxide nanoparticles (Ag-GO), xanthan gum, pigment-xanthan, pigment-xanthan-Ag, pigment-xanthan-GO, and pigment-xanthan-Ag/GO. Xanthan gum displays its characteristic peaks at 3413 cm^−1^ (OH stretching), 2917 cm^−1^ (C-H stretching), 1612 and 1410 cm^−1^ (carboxylate vibrations), 1056 cm^−1^ (C–O stretching), and 1724 cm^−1^ (ester carbonyl), consistent with its typical polysaccharide functional groups^25,26^. When xanthan gum is combined with the pigment, new bands appear at 1548, 1327, 991, and 992 cm^−1^, reflecting functional groups from phytochemical components such as betanin, iso-betanin, and flavonoids. At the same time, original xanthan peaks at 1156, 891, and 789 cm^−1^ disappear^27–29^, indicating strong interactions or masking effects caused by the pigment molecules. When Ag nanoparticles are incorporated into the pigment-xanthan matrix, most characteristic bands of the pigment-xanthan system remain, but several notable spectral changes indicate direct interactions between Ag NPs and functional groups. The disappearance of bands at 1721 cm^−1^ (C = O ester), 1548 cm^−1^ (N-H/C = N), and 1327 cm^−1^ (C–N or phenolic groups) suggests that carbonyl, amine, and phenolic groups participate in reducing Ag⁺ ions and subsequently coordinate to the nanoparticle surface^30,31^. New peaks at 1102 and 829 cm^−1^ indicate the formation of Ag–O and Ag–N coordination bonds and structural rearrangements within the xanthan–pigment network^32^. Shifts in peaks at 1371, 1248, and 590 cm^−1^ reflect changes in C–O, C–O–C^33^, and skeletal bending vibrations, confirming that Ag nanoparticle addition alters the local chemical environment through chelation, electrostatic interactions, and surface adsorption of functional groups^34^. The FTIR spectrum of the pigment-xanthan-GO composite shows the disappearance of several characteristic pigment bands, like the changes observed with Ag nanoparticles, indicating strong interactions between GO and functional groups in the pigment-xanthan matrix. New peaks at 1205, 865, 847, 830, and 470 cm^−1^ suggest that GO’s oxygen-containing groups hydroxyl (–OH), epoxy (–O–), and carboxyl (–COOH) form new bonding or adsorption interactions with pigment molecules and xanthan chains^35,36^. These spectral shifts indicate molecular rearrangement and enhance structural integration, confirming that GO actively modifies the composite structure rather than acting as a passive additive^37^. When both Ag and GO nanoparticles are incorporated into the pigment matrix, several characteristic pigment bands disappear, like the changes observed with Ag or GO individually, indicating that both nanoparticles interact with functional groups in the system. New peaks at 1206, 865, 847, and 470 cm^−1^ reflect combined interactions of GO’s oxygenated groups (–OH, –O–, –COOH) and Ag-ligand bonding with pigment molecules^38^. A distinct peak at 679 cm^−1^, absent in the individual Ag or GO composites, suggests unique structural rearrangements or synergistic effects from the simultaneous presence of both nanoparticles. These spectral changes confirm that the Ag/GO combination induces additional molecular modifications, resulting in a more complex and stabilized hybrid pigment-biopolymer network^39^.

### Raman spectroscopy

**Fig. 3:**
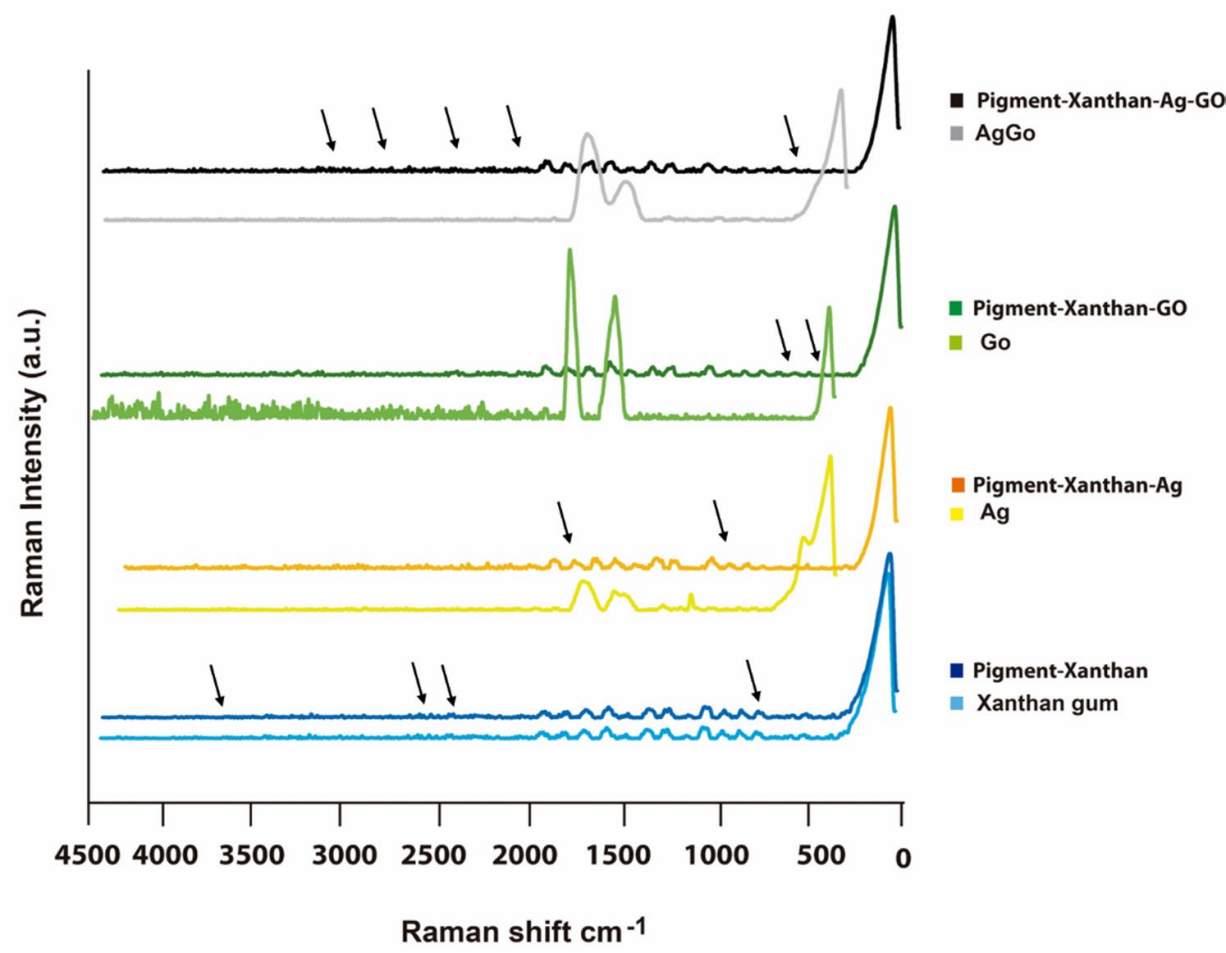
Raman spectra of xanthan gum, pigment-xanthan, silver nanoparticles (Ag), pigment-xanthan-Ag, Graphene oxide (GO) pigment-xanthan-GO, silver-graphene (Ag/GO) and pigment-xanthan Ag/GO. Black arrows indicate the shifted peaks and newly appeared bands resulting from interaction between the components.

Figure 3 illustrates the Raman spectra, showing the interactions between the pigment and xanthan gum, as well as the effects of adding silver nanoparticles, graphene oxide, and their combination with the pigment-xanthan mixture. Xanthan gum shows bands corresponding to skeletal/lattice modes (81 cm^−1^), C–O–C and C–C stretching (544–1109 cm^−1^), C–O–H bending and CH₂ deformations (1200–1418 cm^−1^), and C = O stretching from carboxyl groups (1641–1769 cm^−1^), with overtones in 1872 and 1995 cm^−1^. Upon addition of *Beta vulgaris* pigment, several bands disappear (2690, 1200, 811, 544 cm^−1^)^40^ and new bands appear (3617, 2593, 2485 cm^−1^), reflecting changes in C = O, –OH, and C–O groups. Shifts at 3236, 2644, and 893 cm^−1^ indicate structural alterations and interactions within the pigment-xanthan gum composite^41,42^. With the addition of silver nanoparticles (Ag-NPs), several bands disappear (3617 – OH stretching, 3236 - OH/NH stretching, 2708 & 2644 - CH stretching, 2593 & 2512 –C–H bending), indicating significant changes in molecular vibrations and suggesting interactions between Ag-NPs and the pigment-xanthan gum matrix. A new band at 995 cm^−1^ (C–O/C–C stretching) appears, reflecting the formation of new chemical species or coordination complexes^43^.

Pristine graphene oxide (GO) exhibits the characteristic D (~ 1350 cm^−1^), G (~ 1580 cm^−1^), and 2D (~ 2700 cm^−1^) Raman bands, which correspond respectively to defect-induced vibrations, graphitic sp² carbon stretching, and the overtone of the D band^44^. These peaks reflect the structural disorder and oxidation level typical of GO. When GO is added to the pigment–xanthan gum matrix, most of these characteristic features become suppressed or shift in intensity, indicating strong interactions between GO and the polymer–pigment network. The disappearance of several original Raman bands and the retention of only specific modes (2000, 1893, 1670, 1648, 1411, 1299, 1102, and 76 cm^−1^) suggest changes in GO’s surface chemistry, partial disruption of its sp² domains, and possible formation of hydrogen bonding or electrostatic interactions with xanthan gum and the pigment molecules^45^.

Moreover, after functionalization of GO with silver nanoparticles (Ag-NPs), the characteristic GO peaks, D (~ 1350 cm^−1^), G (~ 1580 cm^−1^), and 2D (~ 2700 cm^−1^) are expected to shift or change in intensity, indicating modifications in defect density and graphitic structure. These effects were considered when interpreting the Ag/GO spectrum^40^. Upon adding the Ag/GO mixture to the pigment-xanthan composite, most original raman bands reappear, while new peaks at 3261–3196 cm^−1^ (–OH stretching), 2854 cm^−1^ (C–H stretching), 2311–2164 cm^−1^ (skeletal vibrations), and 710 cm^−1^ (lattice modes) emerge, suggesting formation of new coordination complexes^41^. Meanwhile, the disappearance of peaks at 3617, 2644, 2485, and 893 cm^−1^ indicates specific structural rearrangements induced by the combined nanoparticles^46^.

### X-ray diffraction (XRD)

**Fig. 4:**
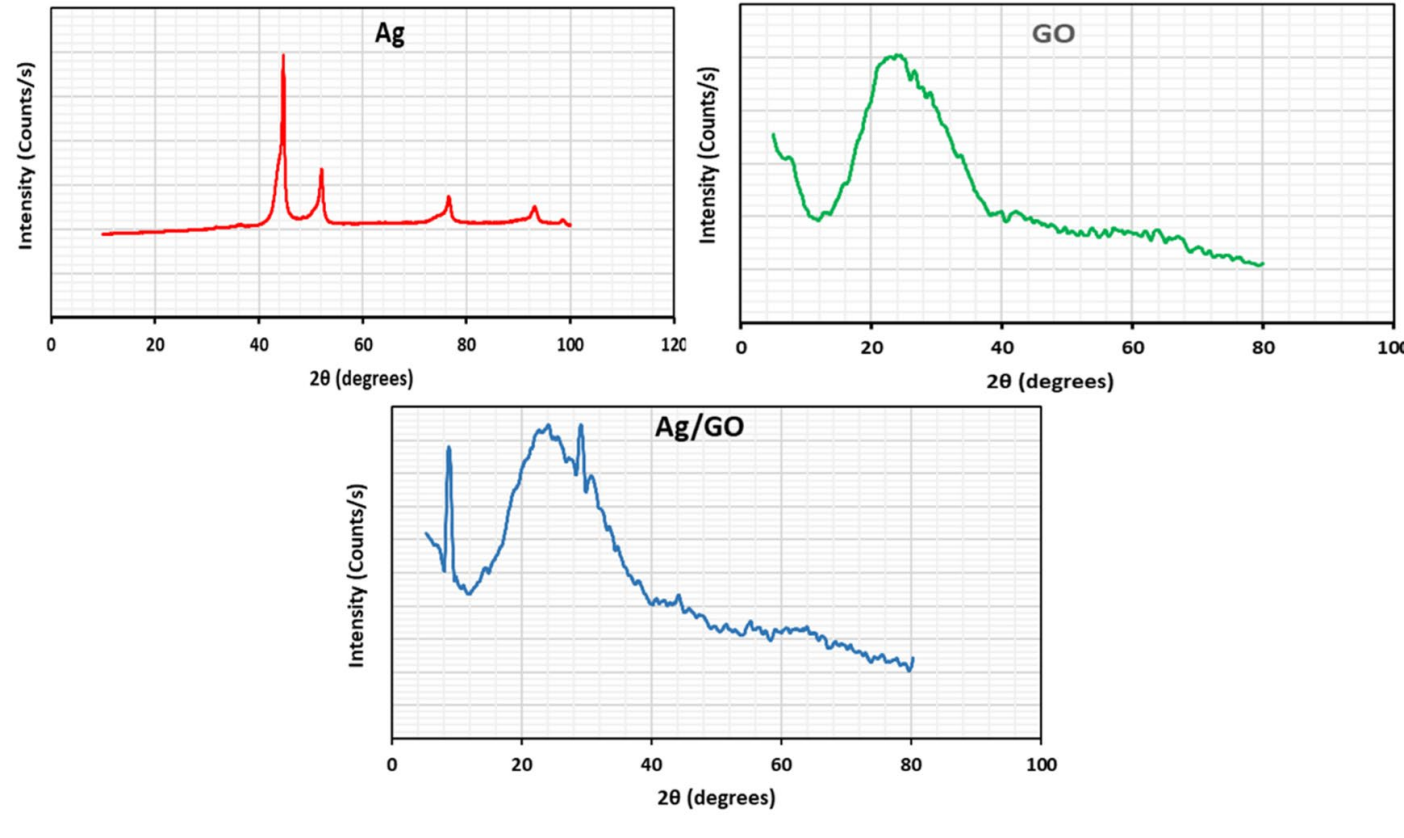
X-ray diffraction (XRD) patterns of silver nanoparticles (Ag), graphene oxide nanoparticles (GO), and (c) silver/graphene oxide nanocomposites (Ag/GO).

The crystalline and phase composition of the synthesized silver nanoparticles (Ag NPs), graphene oxide (GO), and Ag-functionalized GO (GO/Ag) nanostructures were examined using X-ray diffraction (XRD, Fig. 4). The XRD pattern of the Ag NPs showed weak and broadened peaks. Four reflections appeared at approximately 44°, 54°, 77°, and 94°, corresponding to the (111), (200), (220), and (311) planes of the face-centered cubic (FCC) structure of metallic silver, in good agreement with the JCPDS standard card No. 04–0783^47^.The XRD pattern of GO exhibited a broad, amorphous hump with unresolved peaks. Two main features were observed: a low-angle reflection below 10°, attributed to the interlayer ordering of GO sheets, and a broad peak around 30°, corresponding to semi-crystalline graphitic domains^48^. Upon incorporation of Ag into GO, the characteristic GO peaks were significantly reduced and replaced by a broad hump centered at 24.5°, indicative of partial restoration of sp² carbon domains. Only very weak reflections of Ag were observed, consistent with its low loading and high dispersion within the GO matrix. These results confirm the successful integration of Ag NPs into the GO framework while largely preserving the structural integrity of the host material^49^.

### Scanning electron microscopey (SEM)

**Fig. 5:**
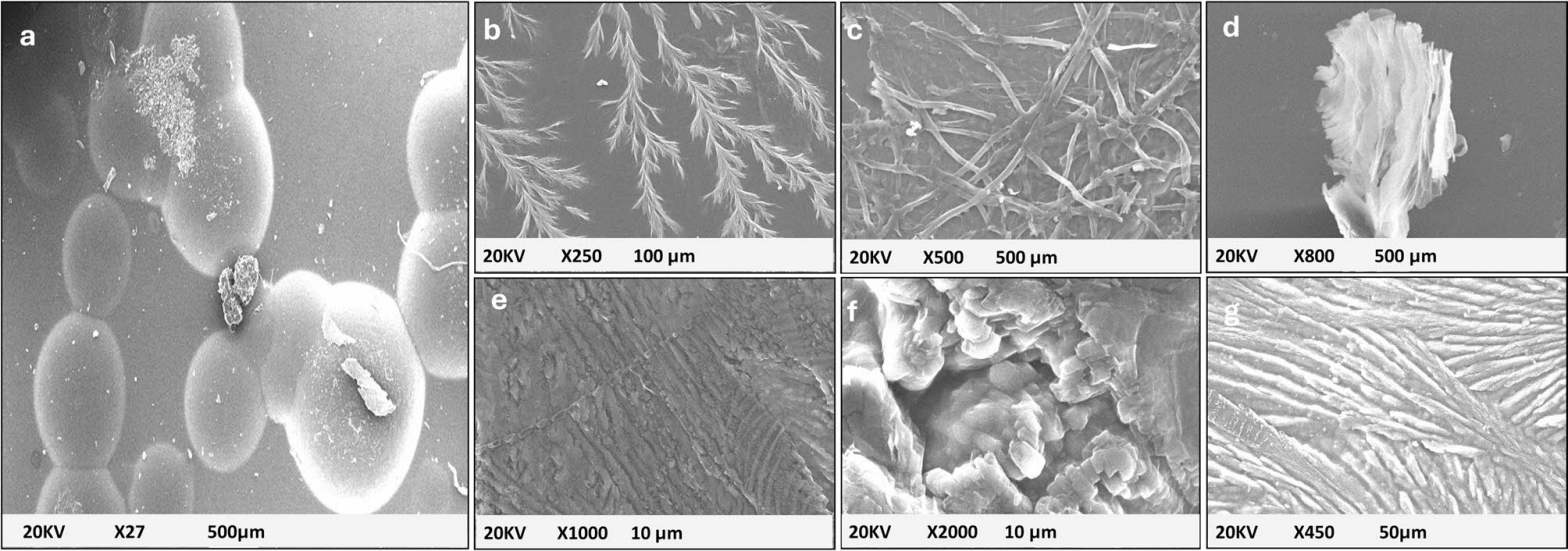
SEM images of (**a**) pigment-xanthan, (**b**) silver-NPs, (**c**) graphene oxide-NPs, (**d**) silver-graphene-NPs, (**e**) pigment-xanthan-Ag-NPs, (**f**) pigment-xanthan-GO-NPs and (**g**) pigment-xanthan-Ag/GO-NPs.

The SEM micrographs of the control and composite samples (Fig. 5) reveal distinct morphologies for each material. Pigment-xanthan gum (Fig. 5a) exhibits smooth, spherical particles, while silver nanoparticles (Ag-NPs, Fig. 5b) display branched, dendritic nanosheets. Graphene oxide nanoparticles (GO-NPs, Fig. 5c) show fibrous, sheet-like structures with wrinkles and folds, whereas silver/graphene oxide nanocomposites (Ag/GO-NPs, Fig. 5d) present layered, sheet-like structures with Ag nanoparticles attached to the GO sheets^50^. The pigment-xanthan-Ag-NPs (Fig. 5e) exhibits linear patterns with cracks, suggesting pigment-silver interactions, while pigment-xanthan-GO-NPs (Fig. 5f) forms compact, porous agglomerates, indicating embedding of the pigment within GO sheets. Finally, the pigment-xanthan-Ag/GO-NPs (Fig. 5g) shows aligned fibrous structures with well-dispersed nanoparticles, confirming the successful integration of all components^51^.

### Transmission electron microscopy (TEM)

**Fig. 6:**
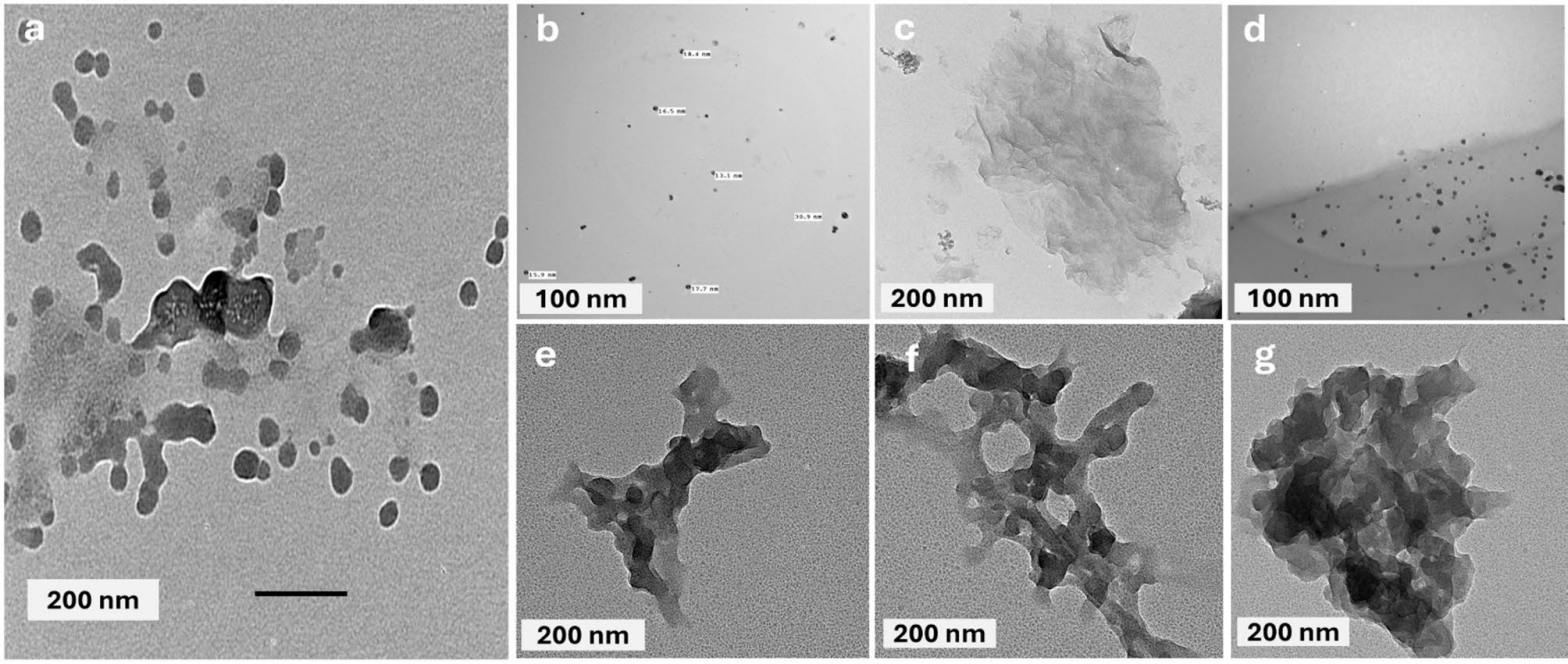
TEM images of (**a**) pigment-xanthan, (**b**) silver-NPs, (**c**) graphene oxide-NPs, (**d**) silver-graphene-NPs, (**e**) pigment-xanthan-Ag-NPs, (**f**) pigment-xanthan-GO-NPs and (**g**) pigment-xanthan-Ag/GO-NPs.

TEM images of the synthesized nanoparticle composites reveal distinct morphologies for each sample. Figure 6a, pigment-xanthan shows aggregated, irregular particles with broad size distribution. Figure 6b, silver (Ag-NPs) appears as well-dispersed dark spots. Figure 6c, graphene oxide (GO-NPs) exhibits thin, wrinkled sheet-like layers. Figure 6d, silver-graphene oxide-NPs composite shows silver-NPs anchored onto graphene sheets, confirming successful integration. Figure 6e, Pigment-xanthan-Ag displays dense particle regions indicating strong interaction with Ag. Figure 6f, pigment-xanthan-GO shows GO sheets with embedded nanoparticles, demonstrating composite formation. Figure 6g, pigment-xanthan-Ag/GO reveals uniformly dispersed nanoparticles stabilized within the matrix, reflecting a controlled and stable nanocomposite formulation. These images indicate that combining Ag and GO with the pigment-xanthan system enhances particle dispersion and may improve stability and functional properties of the composite.

### Elemental composition (EDX)

The elemental composition of the samples was analyzed using energy-dispersive X-ray spectroscopy (EDX). The spectra confirmed the presence of the expected elements, including Ag, C, and O, indicating the successful formation of silver nanoparticles, graphene oxide, and their composites^52^. The detection of Ca, Na, Mg, P, S, Si, and N in the EDX spectra of pigment-containing samples is attributed to the natural mineral and organic constituents of the *Beta vulgaris* pigment. These elements originate from inherent plant biomolecules and trace minerals, supporting the bio-based nature of the pigment rather than contamination^53,54^.

**Table 1:** Elemental analysis of silver nanoparticles (Ag NPs), graphene oxide (GO), pigment-xanthan gum, pigment-xanthan-Ag NPs, pigment-xanthan-GO NPs, and pigment-xanthan-Ag/GO nanocomposites.

Element %	Ag- NPs	GO- NPs	Ag/GO	Pigment-Xanthan	Pigment- Xanthan- Ag - NPs	Pigment- xanthan- GO - NPs	Pigment-Xanthan- Ag/GO -NPs
Ag	92.5	–	25.0	–	0.04	–	0.02
O	7.5	30.0	20.0	1.57	2.33	2.96	2.39
C	70.0	70.0	55.0	96.88	96.81	94.90	96.13
Ca	–	–	–	0.05	0.16	0.05	0.29
Mg	–	–	–	0.10	–	0.16	0.16
P	–	–	–	0.16	0.32	0.27	0.04
S	–	–	–	0.09	0.15	0.14	0.03
Cl	–	–	–	0.22	0.06	0.55	0.18
K	–	–	–	0.51	0.08	0.95	0.57
Si	–	–	–	–	0.04	–	0.03
N	–	–	–	0.18	–	–	–
Na	–	–	–	0.24	–	–	0.16

### UV spectroscopy

**Fig. 7:**
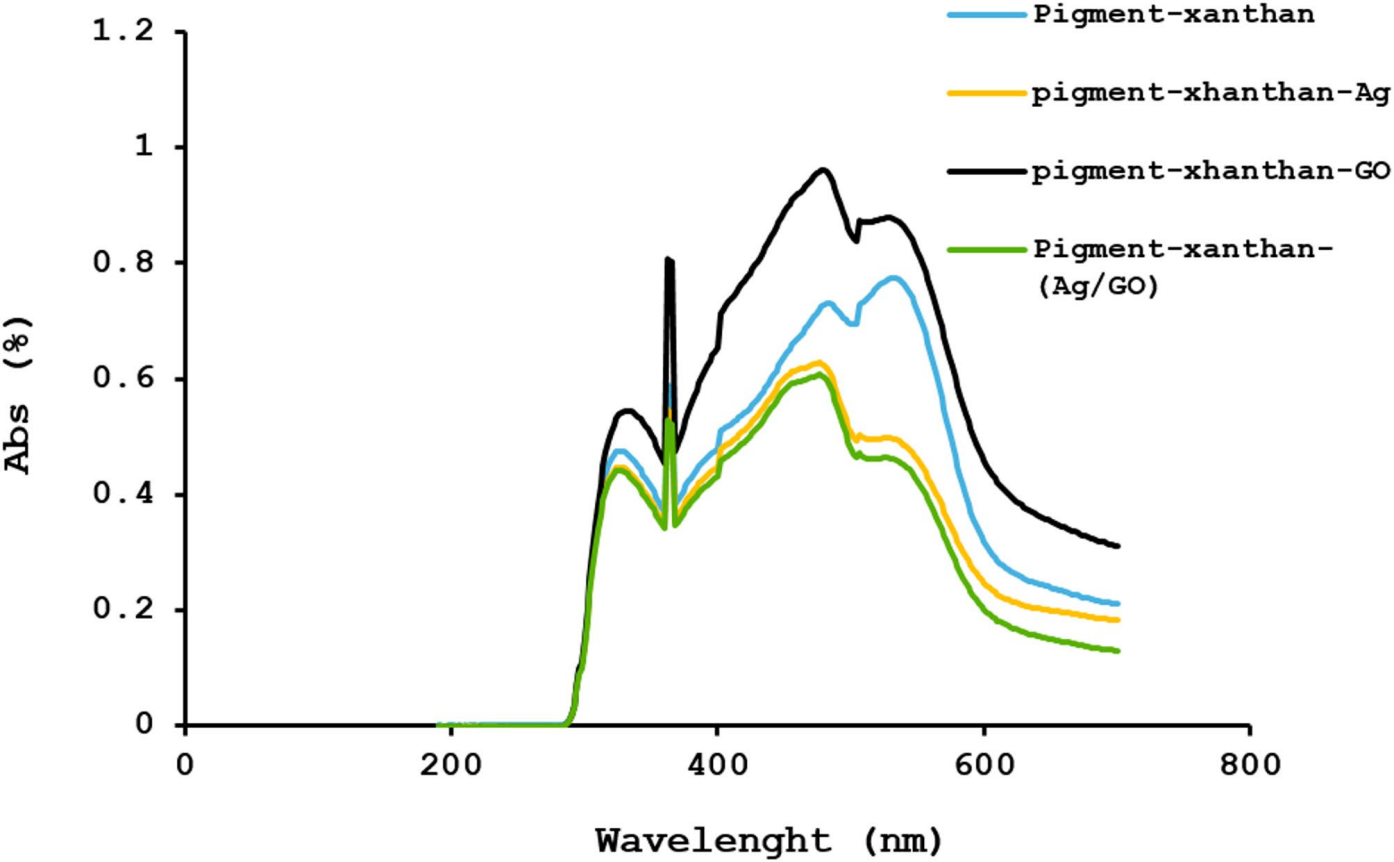
Absorption spectrum of pigment-xanthan, pigment- xanthan-Ag, pigment- xanthan-GO, and pigment- xanthan-Ag/GO.

Figure 7 shows the UV–Vis spectra of Beta vulgaris extract and its composites. All the samples exhibit a strong absorption peak at ~ 350 nm, attributed to betalain pigments, specifically betaxanthins, indicating retention of their characteristic optical properties^55^. In the pigment-xanthan-Ag nanoparticles (orange spectrum), a distinct peak at 400–450 nm corresponds to the surface plasmon resonance (SPR) of Ag nanoparticles, reflecting efficient nanoparticle formation^56^. The pigment-xanthan-graphene oxide (GO, black spectrum) shows a broader peak at 500–600 nm, related to π–π* transitions of sp² carbon in GO, with broadening due to oxygenated groups and layered structure^57^. The pigment-xanthan-Ag/GO composite (green spectrum) displays features of both Ag and GO: the SPR peak of Ag at 400–450 nm and the broad GO-related band at 500–600 nm, with slight shifts suggesting electronic interactions between Ag and GO while preserving their individual optical characteristics^58^.

### Fluorescence spectroscopy

**Fig. 8:**
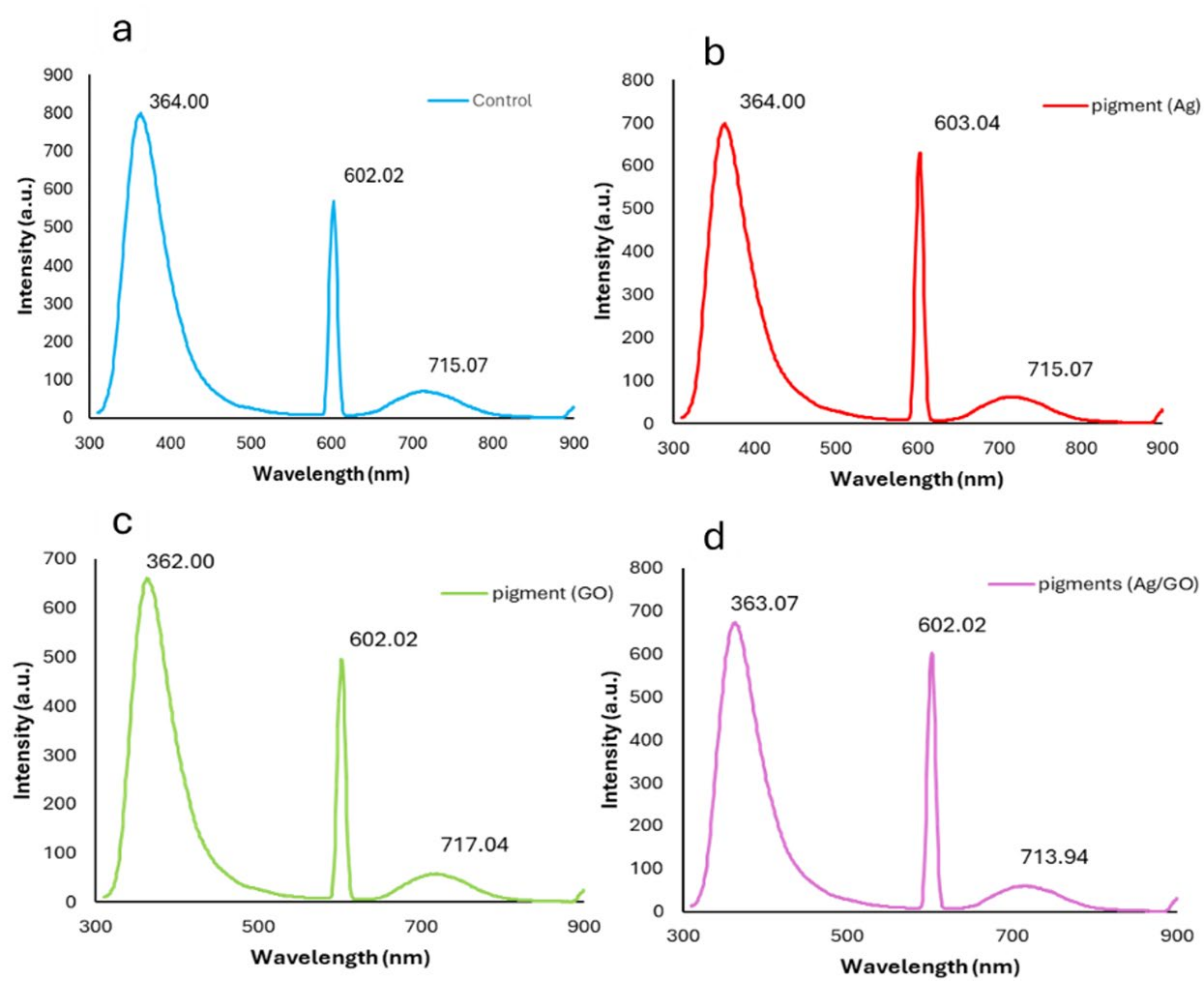
Fluorescence spectroscopy of (**a**) pigment without metals, (**b**) pigment-Ag-NPs, (**c**) pigment-GO-NPs, (**d**) pigment-Ag/GO-NPs.

The fluorescence spectra show different wavelength responses for control and treated samples (Fig. 8). The absorption peaks visible at 364 nm, 602 nm, and 715 nm in the pigment sample correspond to natural pigments present in *Beta vulgaris*, such as betalains^59^. For the treated samples silver (Ag), graphene oxide (GO) nanoparticles, and the composite (Ag/GO) the spectra show a similar pattern, with peaks around 360 nm and 600 nm, but differences in intensity and peak sharpness, indicating changes due to doping and interactions with nanoparticles. This suggests that nanoparticle doping affects the light absorption and emission properties of the material, enhancing its optical behavior^60^.

### Pigments Stability

**Fig. 9:**
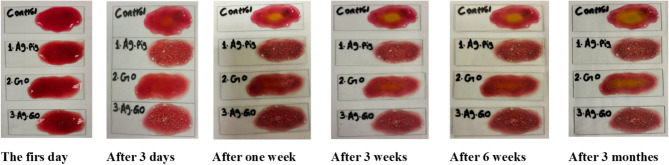
Stability of pigments: Control (pigment-xanthan), 1 Ag (pigment-xanthan-Ag), 2 GO (pigment xanthan- GO), and 3 Ag/GO (pigment xanthan- Ag/GO).

**Natural pigments** are sensitive to light, heat, and oxygen, which can lead to degradation and color loss (control sample). However, its stability can be significantly improved by using metal nanoparticles such as silver (1 Ag) and graphene oxide (2 GO), or a mixture of both (3 Ag & GO). These nanoparticles act as protective agents or catalysts, shielding natural pigments from environmental factors. Silver nanoparticles can enhance pigments’ resistance to oxidative damage and reduce photodegradation, while graphene oxide helps stabilize the pigment by creating a controlled microenvironment, thereby preserving its color for longer periods (Fig. 9).

### Antimicrobial activity

**Fig. 10:**
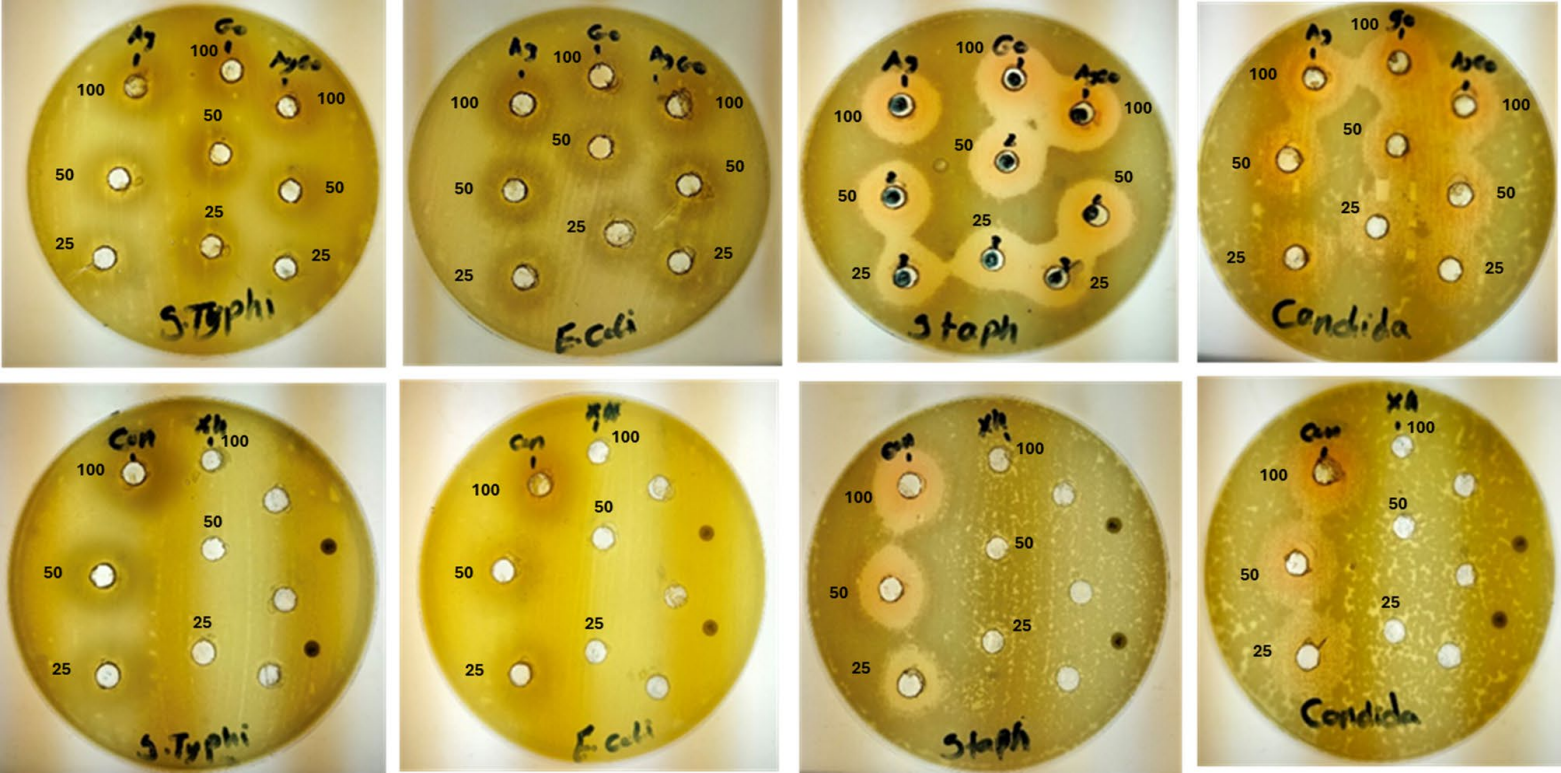
Antimicrobial activity of Pigment-xanthan (Con.), pigment- xanthan Ag-NPs (Ag), pigment-xanthan-GO-NPs (GO), pigment-xanthan-Ag/GO (Ag-GO), and xanthan gum (Xh.) tested at varying concentrations (100, 50 and 25 mg/ml).

The antimicrobial efficacy of pigment-xanthan gum, pigment-xanthan-silver NPs, pigment-xanthan-graphene oxide NPs (Pig-GO), and pigment-xanthan-Ag/GO NPs was evaluated against Gram-positive (*S. aureus*), Gram-negative (*E. coli*, *S. typhi*), and *C. albicans* at 25, 50, and 100 mg/mL (Table 2; Fig. 10). All formulations exhibited concentration-dependent increases in inhibition zones, with nanocomposites showing significantly higher antimicrobial activity than the control pigment (*p* < 0.05). Inhibition zones ranged from 7 to 21 mm at 25 mg/mL, 10–29 mm at 50 mg/mL, and 12–30 mm at 100 mg/mL. *S. aureus* consistently showed the largest zones (up to 30 mm), while *C. albicans* showed the smallest (12 mm), reflecting differences in cell wall structure. The superior activity of pigment-xanthan-Ag/GO is attributed to the synergistic mechanism of Ag⁺ ions and GO sheets: Ag⁺ disrupts cell membranes and interacts with intracellular components, while GO facilitates reactive oxygen species (ROS) generation and physical membrane rupture^61,62^. Nanoparticle size and Beta vulgaris bioactive compounds further contribute to efficacy. Additionally, Ag-NPs and GO enhance pigment stability by scavenging ROS, preventing oxidative degradation, and providing antioxidant and UV protection^63–65^. Overall, the results demonstrate that antimicrobial activity is both concentration- and microorganism-dependent, with potential applications in food, cosmetics, and textile industries.

**Table 2:** Antimicrobial activity of pigment-xanthan-Ag-NPs, pigment- xanthan- GO-NPs, pigment- xanthan-Ag/GO, pigment-xanthan and xanthan gum, tested at varying concentrations (100, 50 and 25 mg/ml).

Sample		Gram negative		Gram positive		Fungi
	Con. mg/ml	E. coli	S. typhi	S. aureus	Candida	
		Inhibition zone (mm)				
Pigment-xanthan-Ag	100	25.33^bc^±0.58	20.00^ef^±1.00	28.67^b^±0.58	12.00^ab^±0.00	
	50	26.00^b^±1.00	22.00^cd^±0.00	25.00^d^±1.00	11.67^ab^±0.58	
	25	24.33^d^±0.58	19.00^f^±0.00	24.33^d^±1.15	10.00^cd^±0.00	
Pigment- xanthan- GO-NPs	100	25.00^cd^±0.00	23.00 ^c^±0.00	30.00 ^a^ ±1.15	11.00^bc^±0.00	
	50	26.00^b^±0.00	22.67 ^c^±0.58	29.00^ab^±0.00	10.00^cd^±0.00	
	25	25.00^cd^±1.00	21.00 ^de^±1.73	25.00^d^±0.00	10.00^cd^±0.00	
Pigment- xanthan-Ag/GO	100	27.00^a^±0.00	21.67^cd^±0.58	29.67 ^ab^±1.53	12.33^a^±0.58	
	50	25.00^cd^±0.00	20.67^de^±1.15	24.00^d^±0.00	11.00^bc^±1.00	
	25	23.00^e^±0.00	7.00 ^g^±1.00	22.00^e^±0.00	8.33^a^±1.15	
Pigment- xanthan-	100	25.33^bc^±0.58	28.00^a^±0.00	30.00^a^±0.00	9.00^a^±1.73	
	50	25.00^cd^±0.00	26.00^b^±0.00	27.33^c^±1.15	10.00^cd^±0.00	
	25	19.00^f^±0.00	21.00 ^de^±1.73	20.00^f^±0.00	7.67^de^±0.58	
Xanthan gum		n.d.	n.d.	n.d.	n.d.	
LSD		0.84	1.53	1.22	1.23	

All experiments were performed in triplicate, and the data are presented as mean ± SD. Different letters within the same column indicate statistically significant differences at *p* ≤ 0.05.

### Minimum inhibitory concentrations

**Fig. 11:**
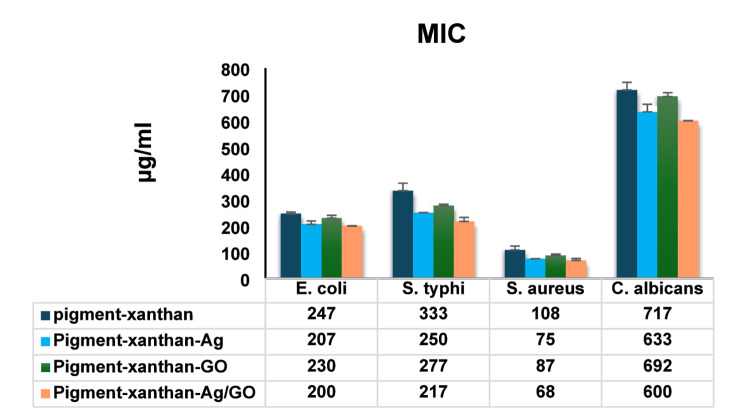
Minimum inhibitory concentrations (MICs) of pigment–xanthan, pigment-xanthan-Ag NPs, pigment-xanthan-GO NPs, and pigment-xanthan-Ag/GO nanocomposites against selected microbial strains.

Figure 11 shows the minimum inhibitory concentrations (MICs) of the pigment-xanthan, pigment- xanthan-Ag, pigment-xanthan-GO, and pigment-xanthan-Ag/GO nanocomposites against Gram-negative bacteria (*E. coli*,* S. typhi*), Gram-positive *S. aureus*, and *Candida albicans*. The control pigment exhibited moderate activity, with MICs of 247–333 µg/mL for Gram-negative bacteria, 108 µg/mL for *S. aureus*, and 717 µg/mL for *C. albicans*. Pig-Ag showed enhanced antimicrobial effects, reducing MICs to 207–250 µg/mL for Gram-negative strains, 75 µg/mL for *S. aureus*, and 633 µg/mL for *C. albicans*, highlighting the potency of silver nanoparticles. Pig-GO also improved activity (230–277 µg/mL for Gram-negative, 87 µg/mL for *S. aureus*, 692 µg/mL for *C. albicans*), reflecting GO’s role in membrane disruption and pigment stabilization. The Pig-Ag-GO nanocomposite was the most effective, with the lowest MICs: 200 µg/mL (*E. coli*), 217 µg/mL (*S. typhi*), 68 µg/mL (*S. aureus*), and 600 µg/mL (*C. albicans*), demonstrating a synergistic effect between silver and graphene oxide that enhances antimicrobial potency and potentially improves pigment stability and bioavailability^66^.

## Conclusion

In conclusion, this study demonstrates the green synthesis of silver (Ag) and graphene oxide (GO) nanoparticles and their combination with Beta vulgaris (beetroot) extract using chitosan and Xanthan gum as stabilizing polymers. The integration of Ag and GO nanoparticles with the bioactive phytochemicals in Beta vulgaris enhanced the stability of key pigments, including anthocyanins and betacyanins, by preventing oxidative degradation and providing antioxidant protection. The antimicrobial evaluation revealed that the pigment nanoparticle composites exhibited concentration-dependent activity, with Pigment-xanthan-Ag/GO showing the highest efficacy. This enhanced antimicrobial effect is attributed to the synergistic mechanism of Ag⁺ ions and GO sheets, where Ag⁺ disrupts bacterial cell membranes and interacts with intracellular components, while GO facilitates reactive oxygen species (ROS) generation and physical membrane rupture, together promoting efficient microbial inactivation. Among the tested strains, *S. aureus* was most susceptible, Gram-negative bacteria showed moderate susceptibility, and C. albicans required higher concentrations, reflecting cell wall differences. Incorporation of Ag-NPs and GO enhanced pigment stability, UV resistance, and functional properties, enabling the development of bioactive, plant-based formulations for food, cosmetics, and biomedical applications. Future studies will assess cytotoxicity and biocompatibility to support safe application of these nanocomposites.

## Supplementary Information

Below is the link to the electronic supplementary material.

Supplementary Material 1

Supplementary Material 2

## Data Availability

The authors confirm that the data supporting the findings of this study are available within the article and its supplementary materials.
