# Supplementary figures and images for: Novel enhancement of stability and antimicrobial activity of beetroot pigment nanocomposites via graphene oxide and silver nanoparticles
Source: Sci Rep. 2026 Mar 26;16:10478. doi: 10.1038/s41598-026-42211-w (PMC13031538; doi:10.1038/s41598-026-42211-w)

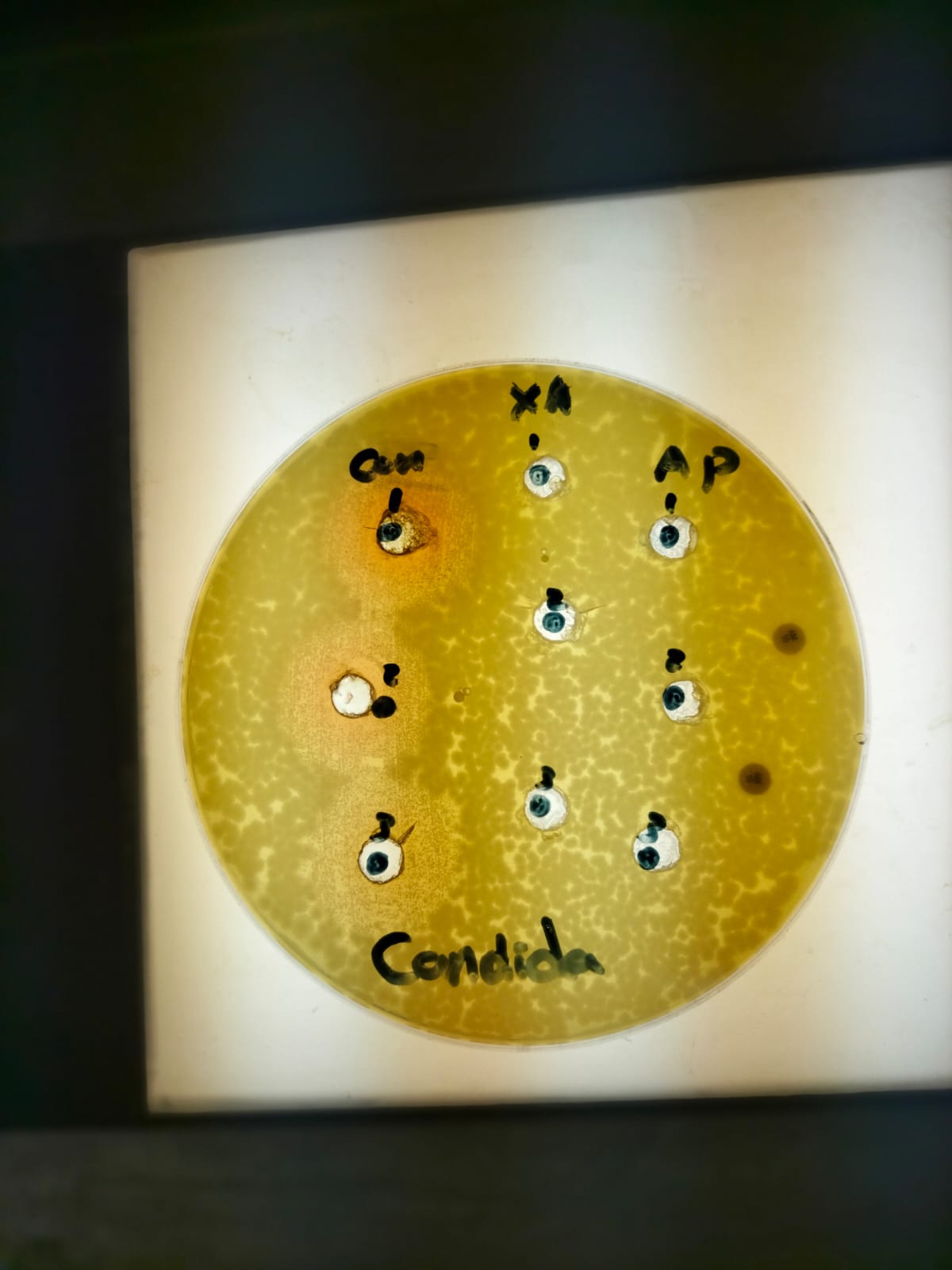

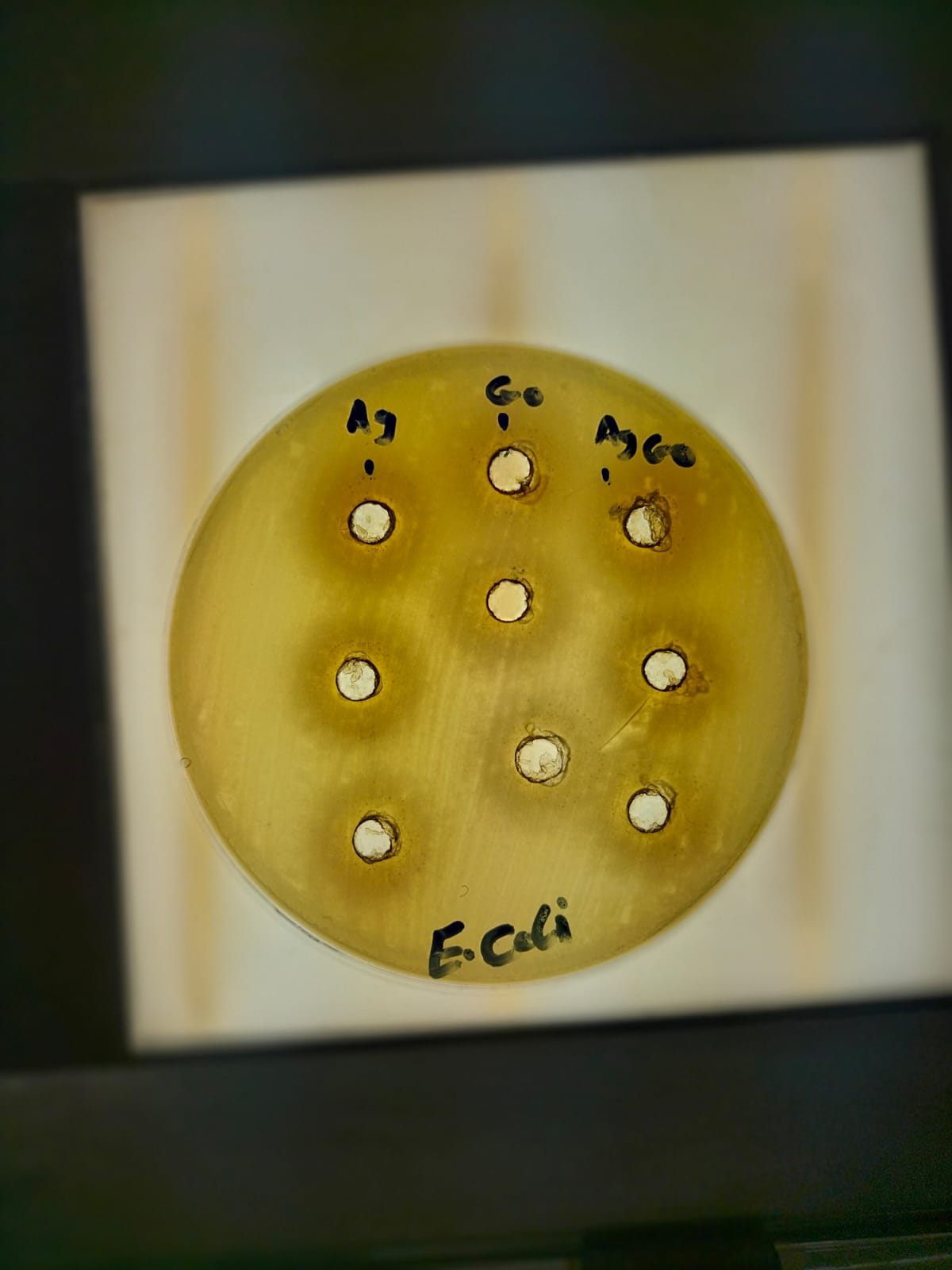

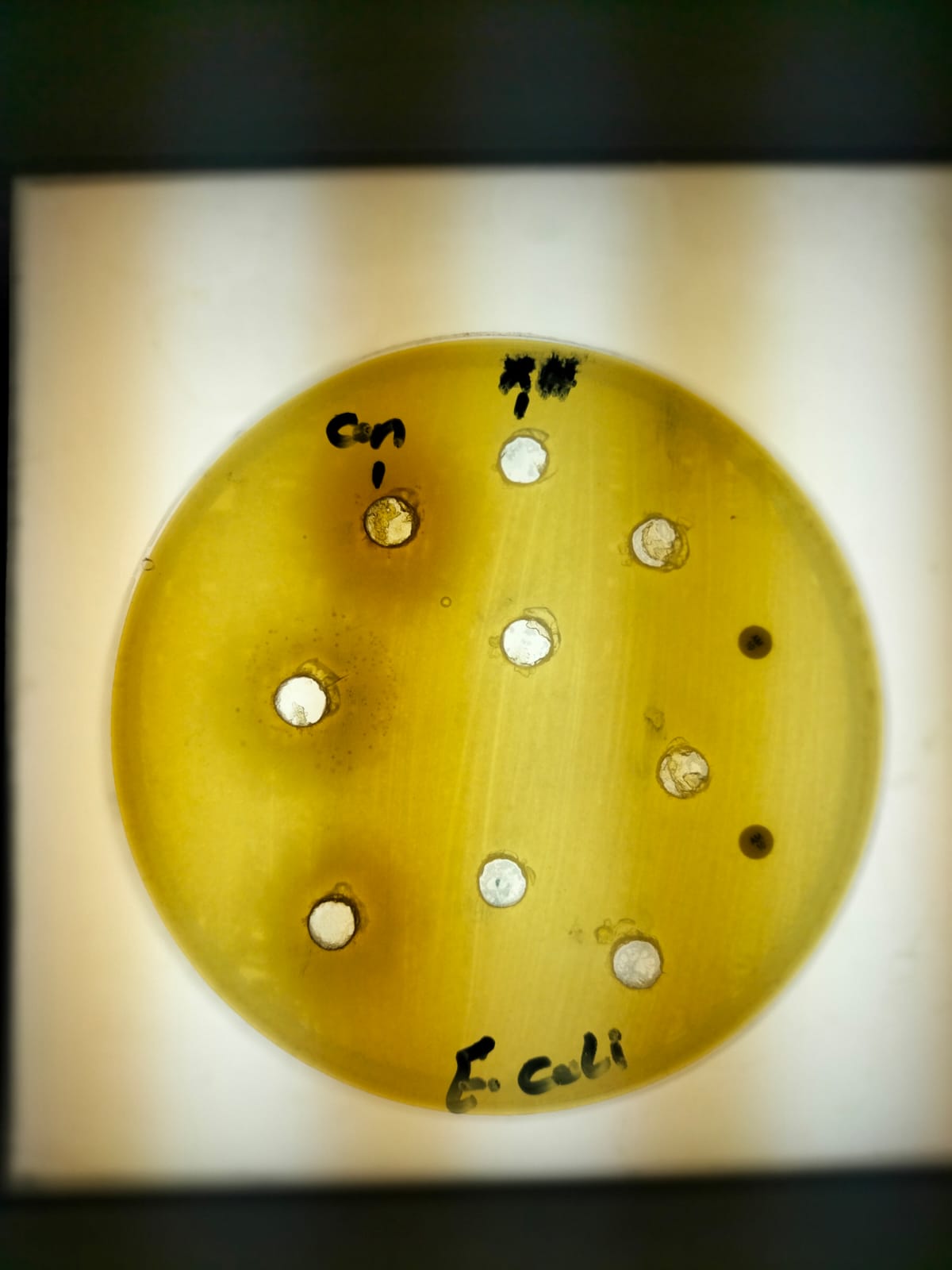

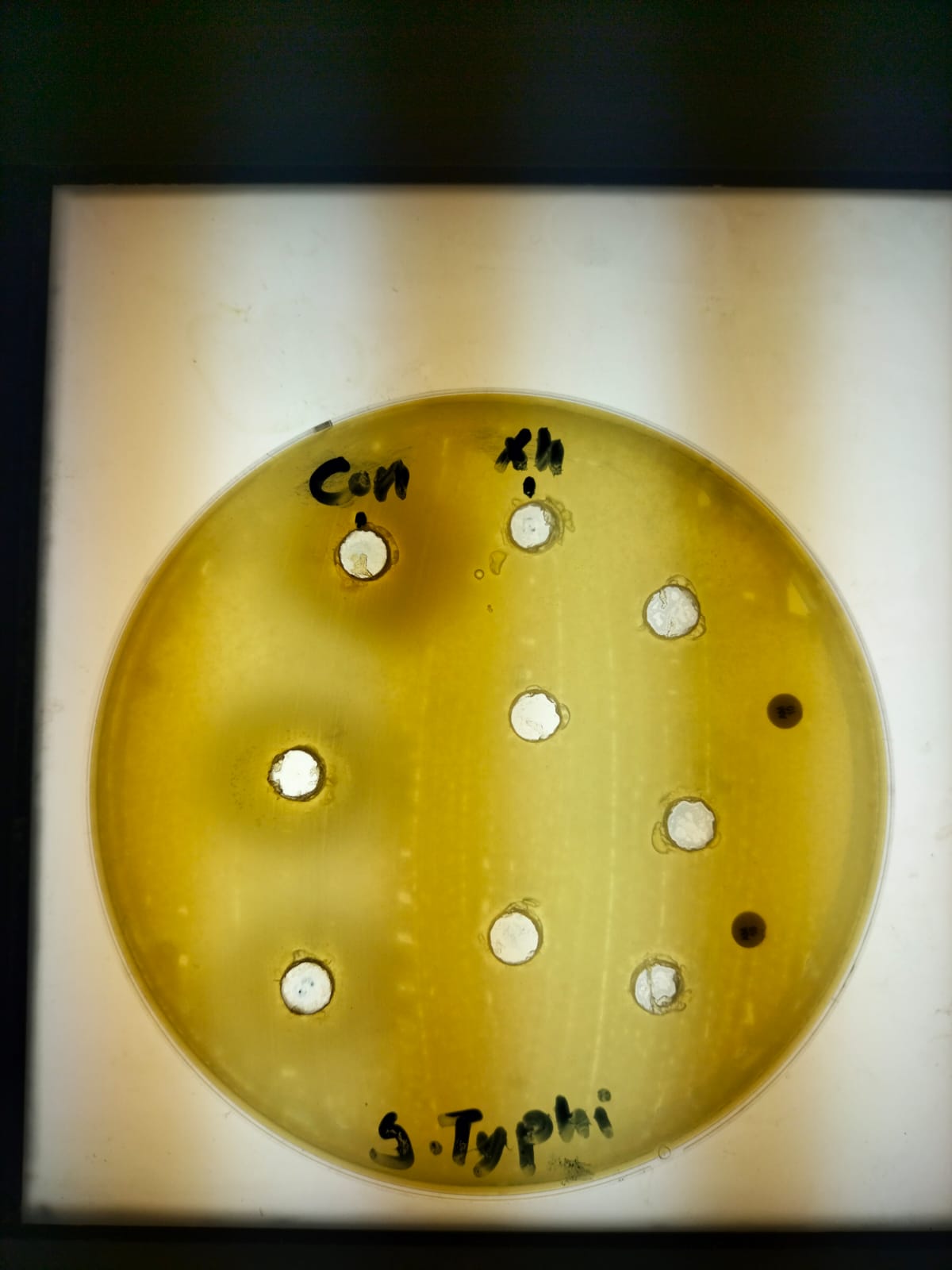

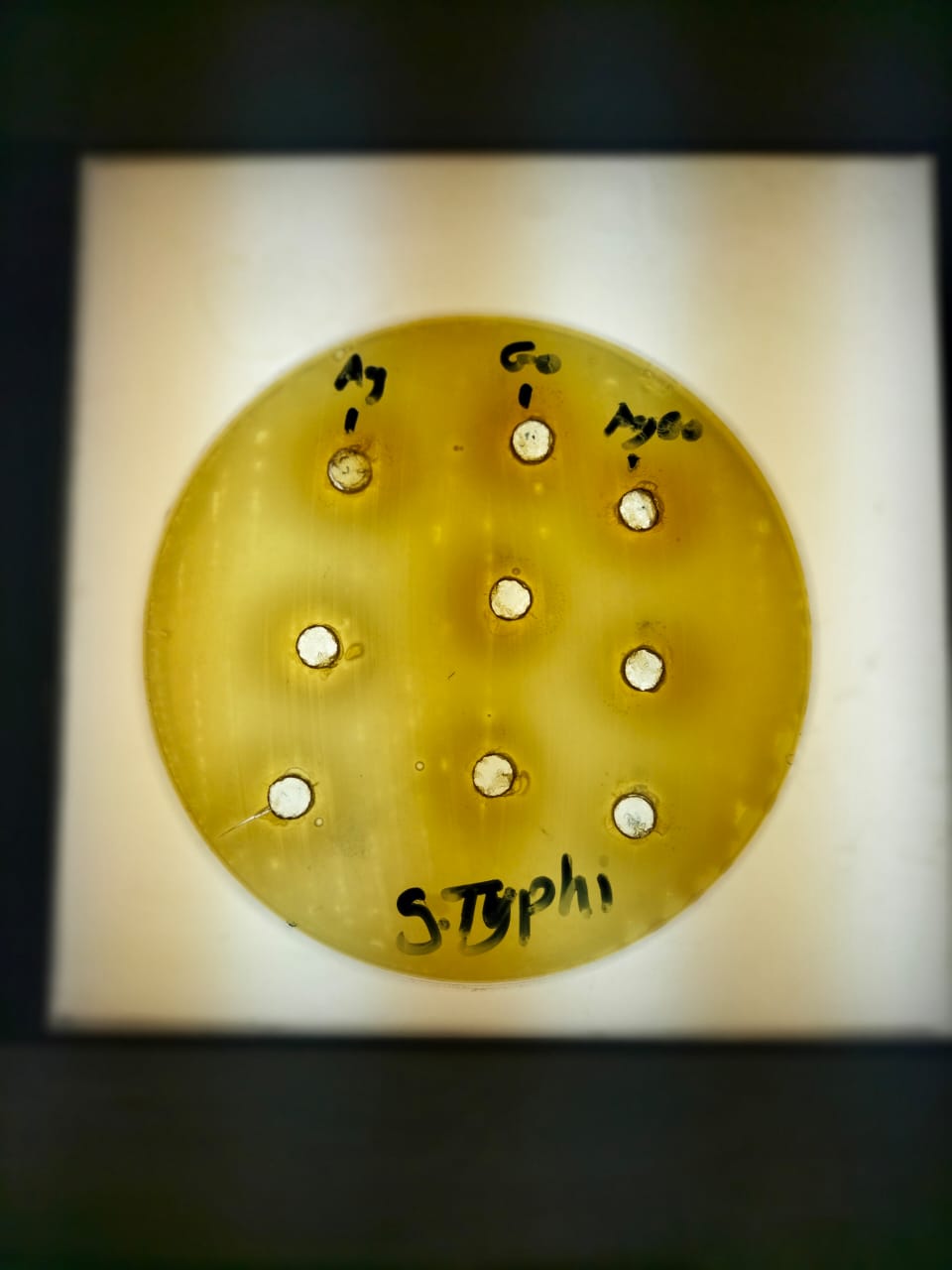

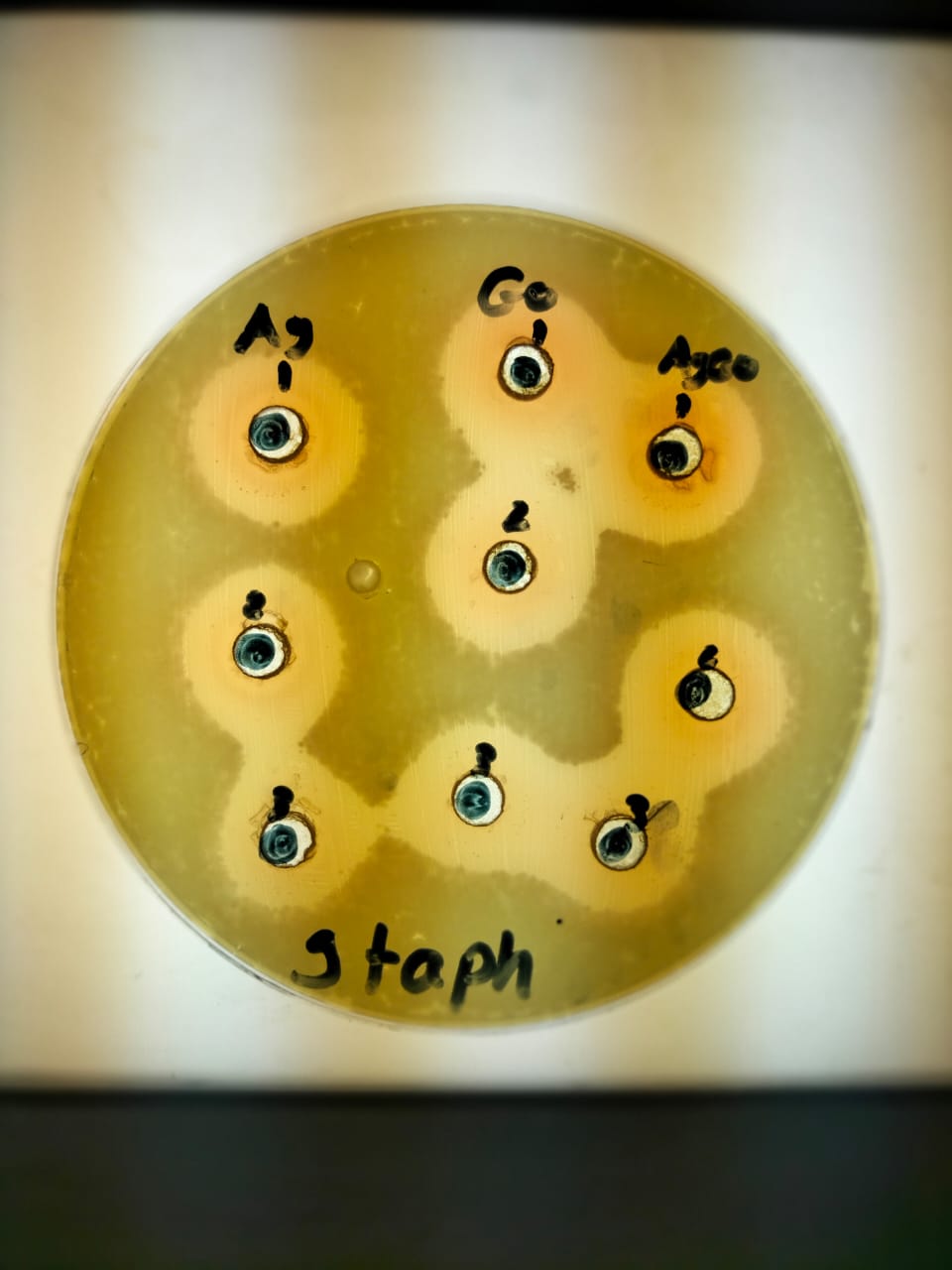

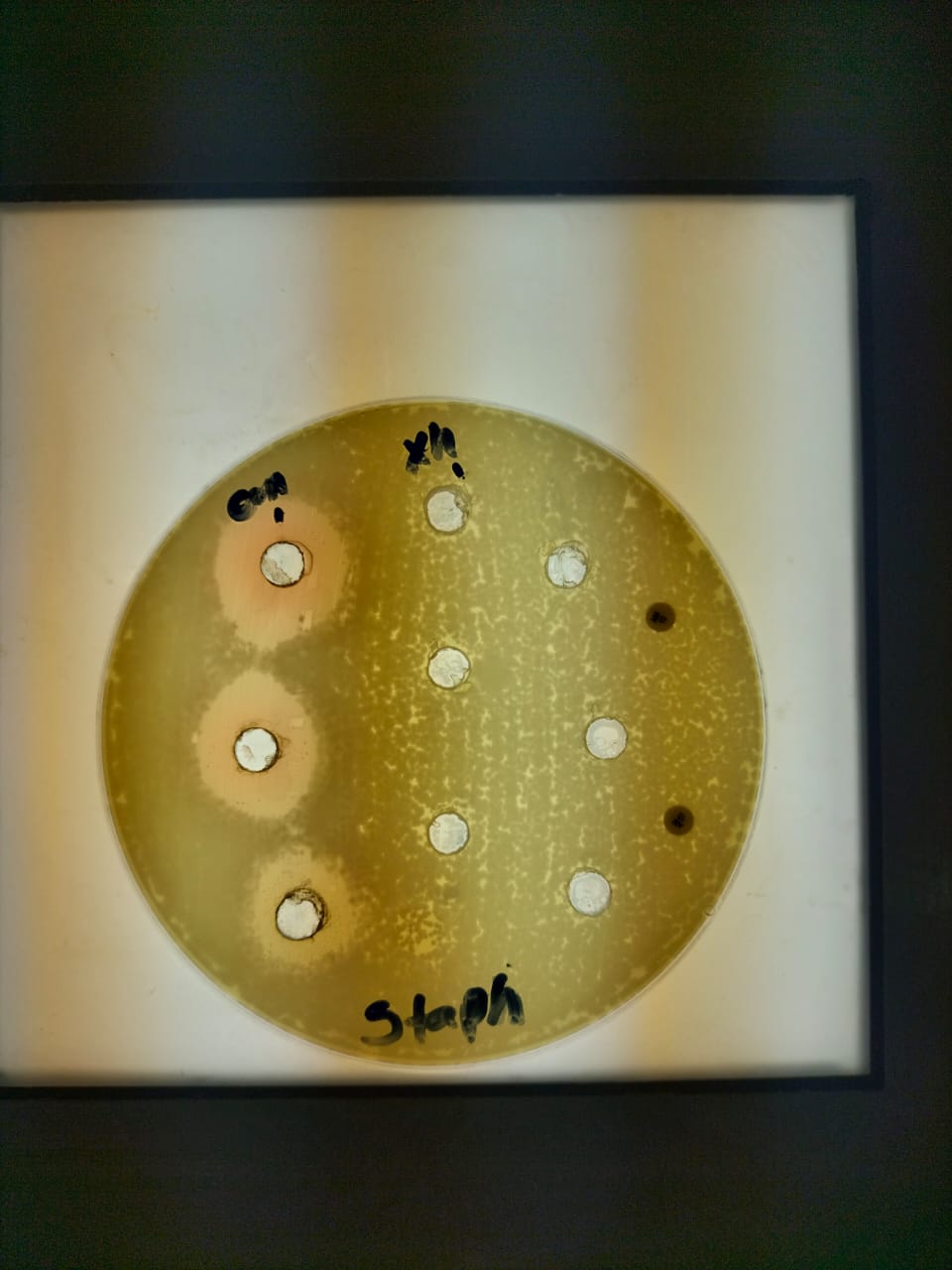

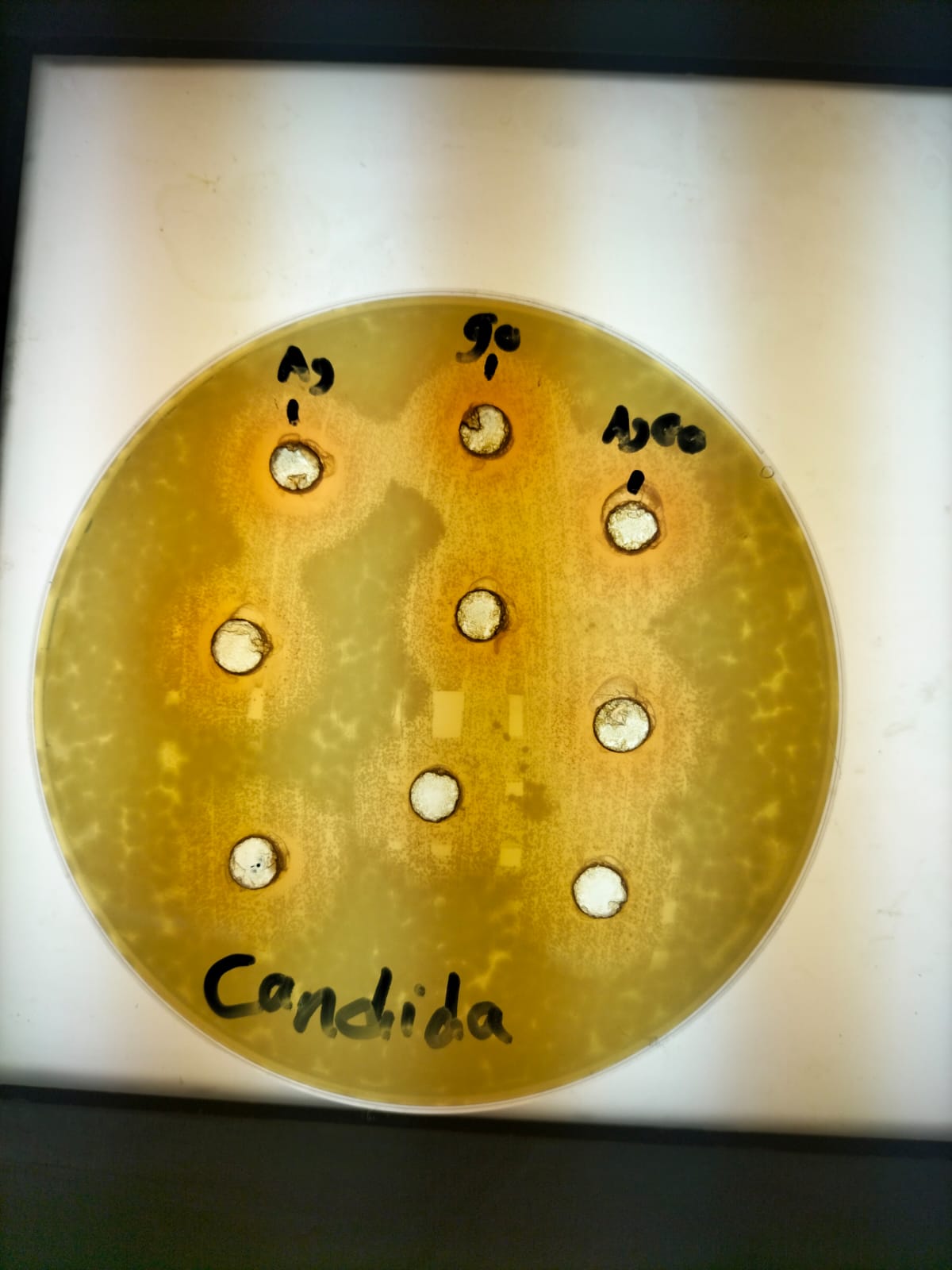


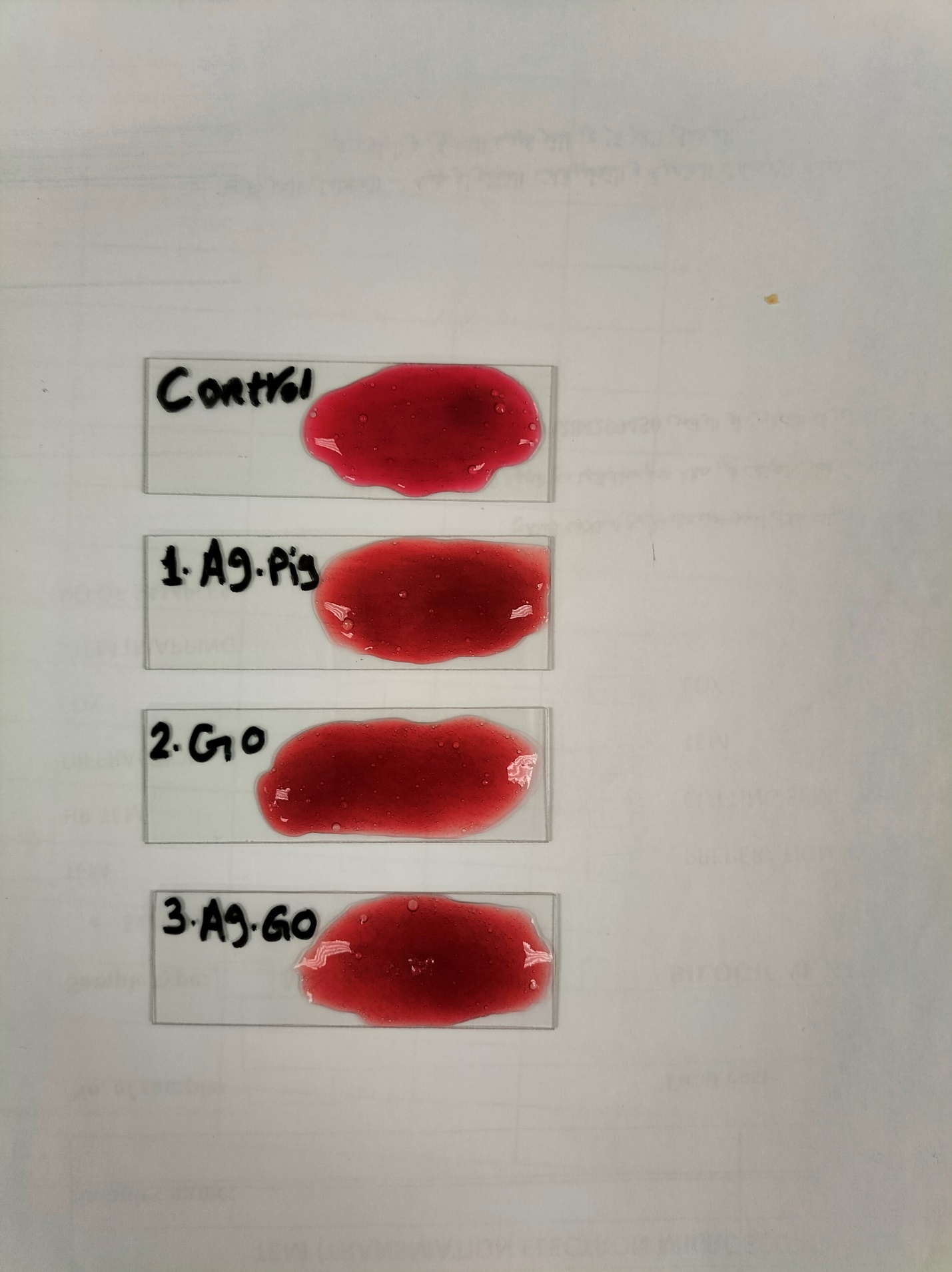

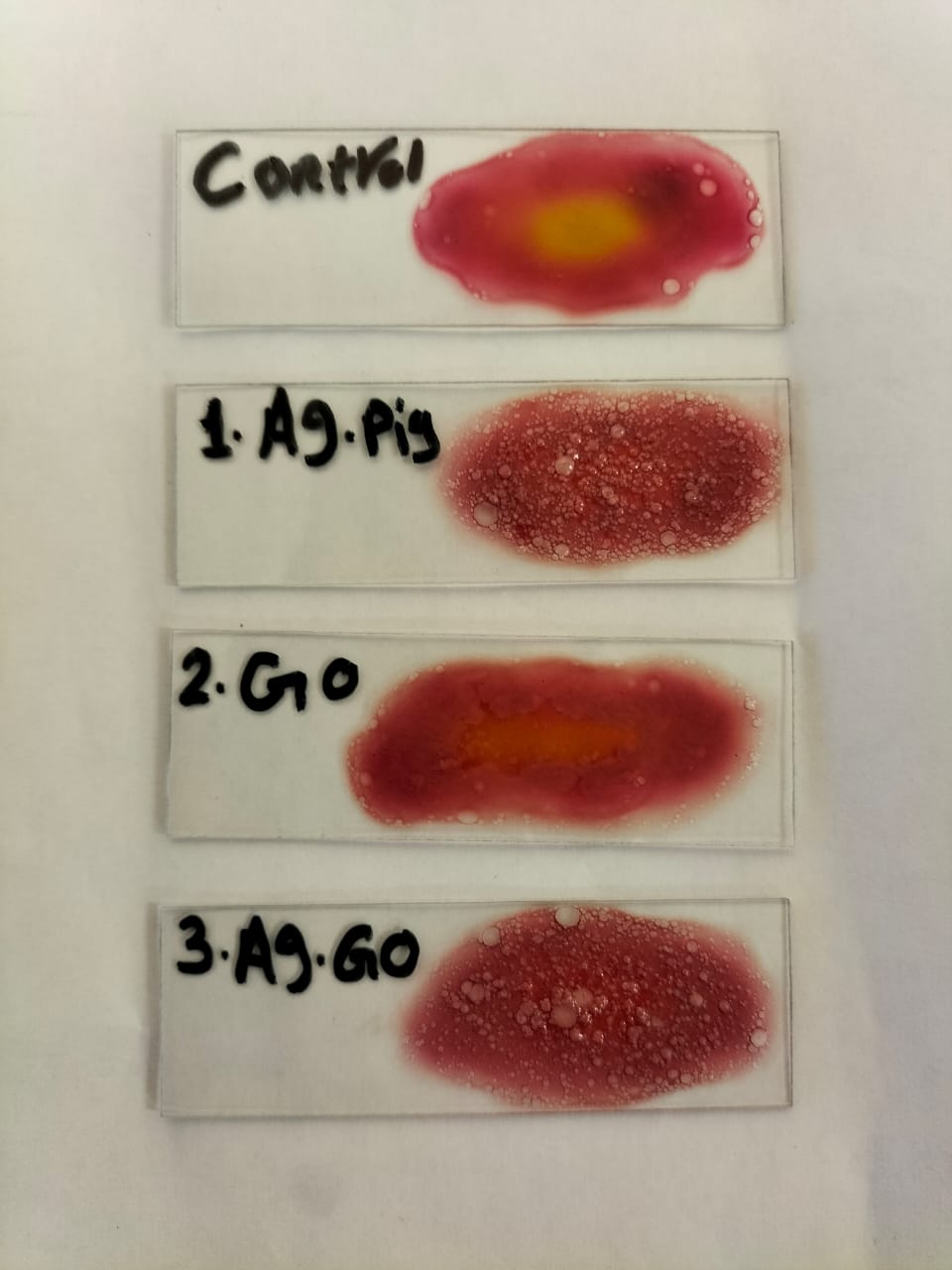

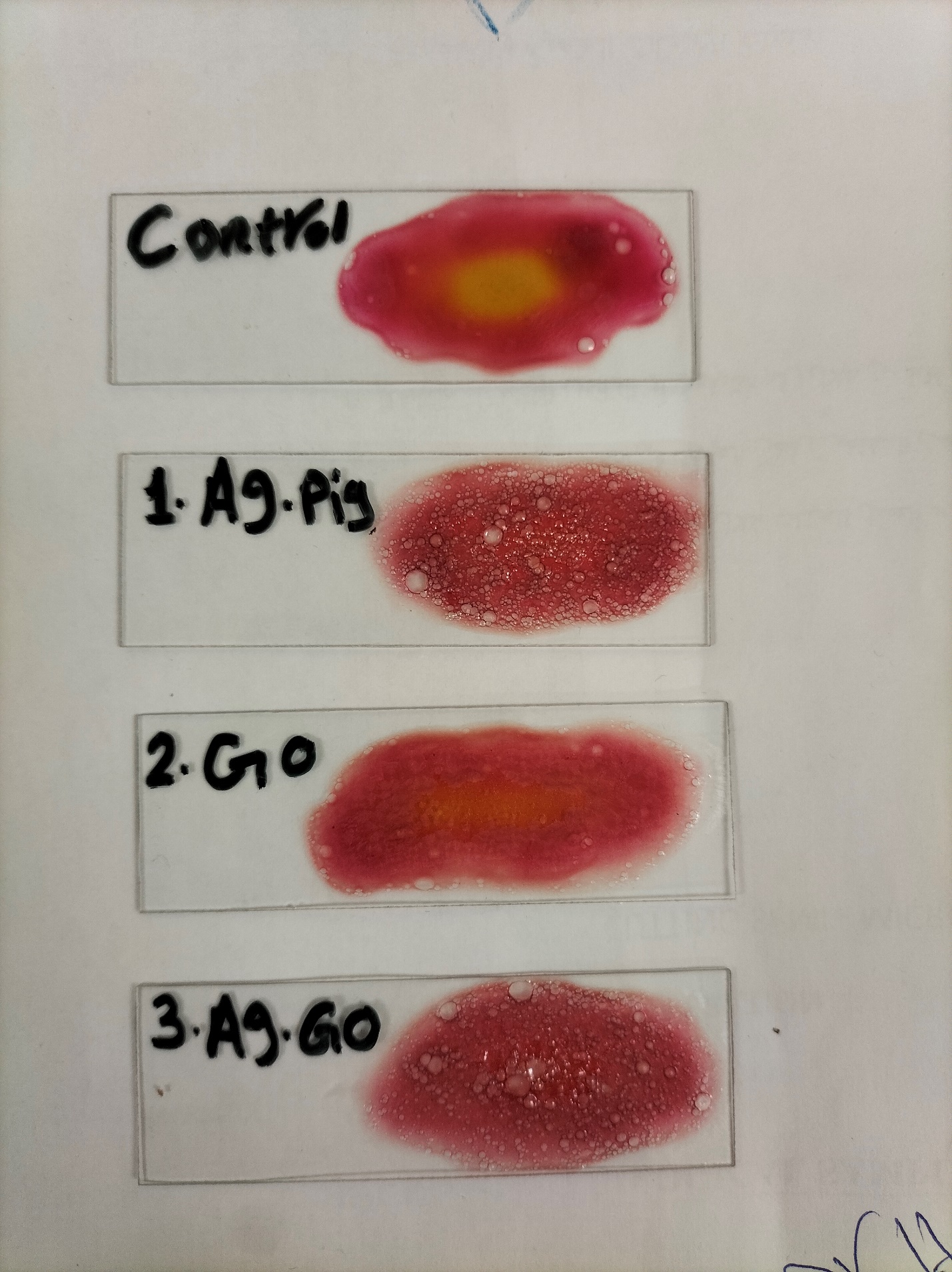

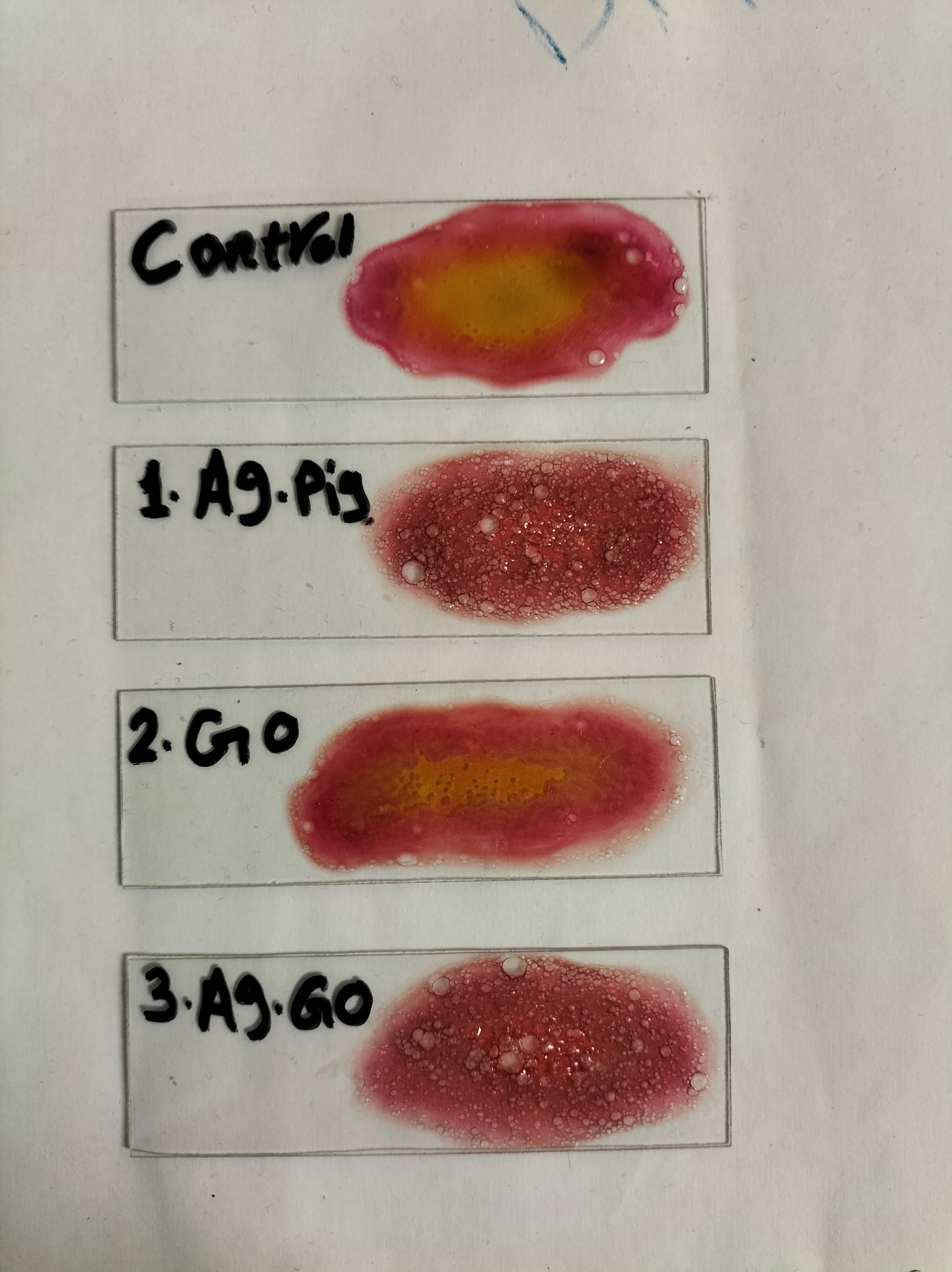

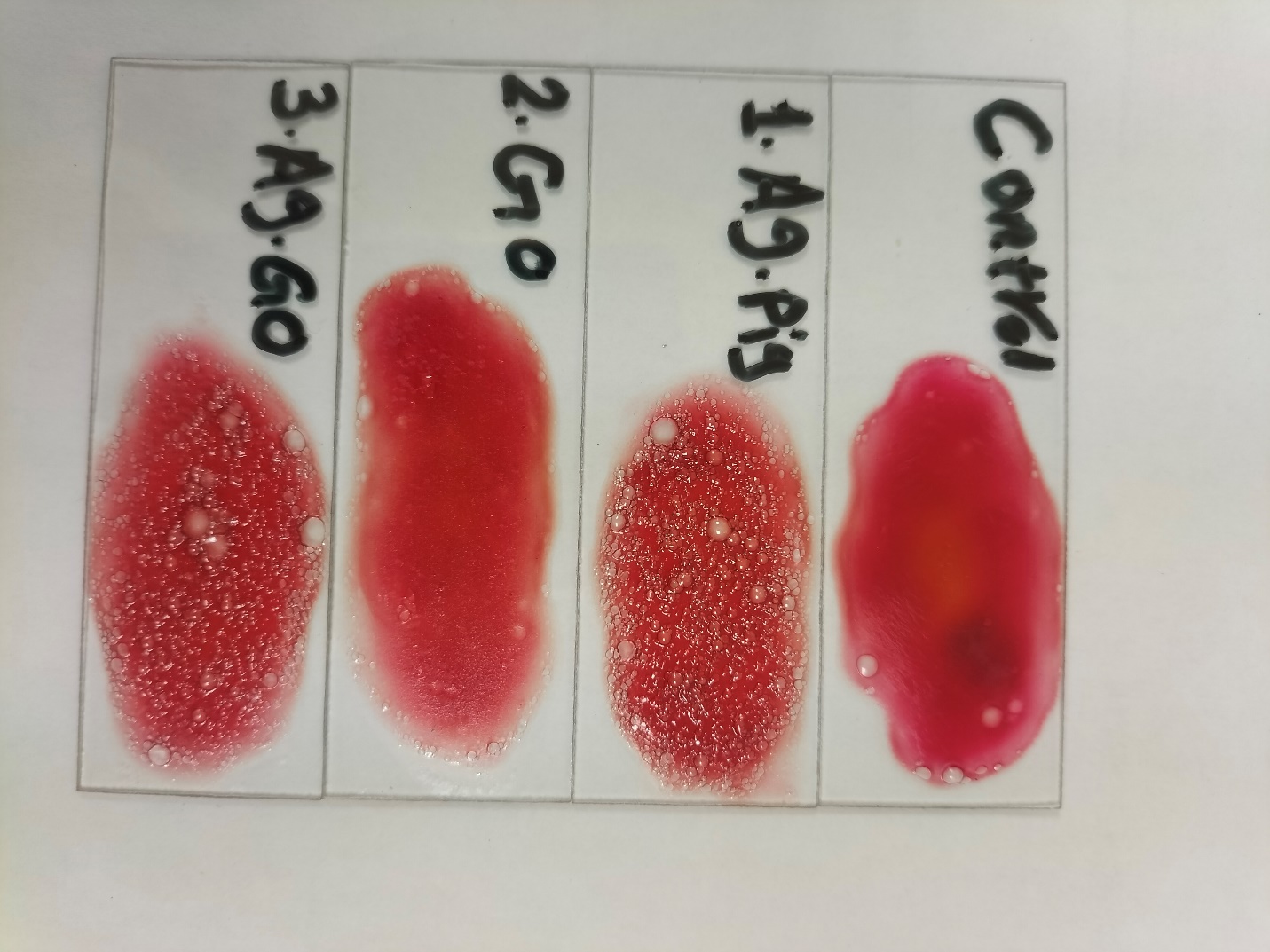

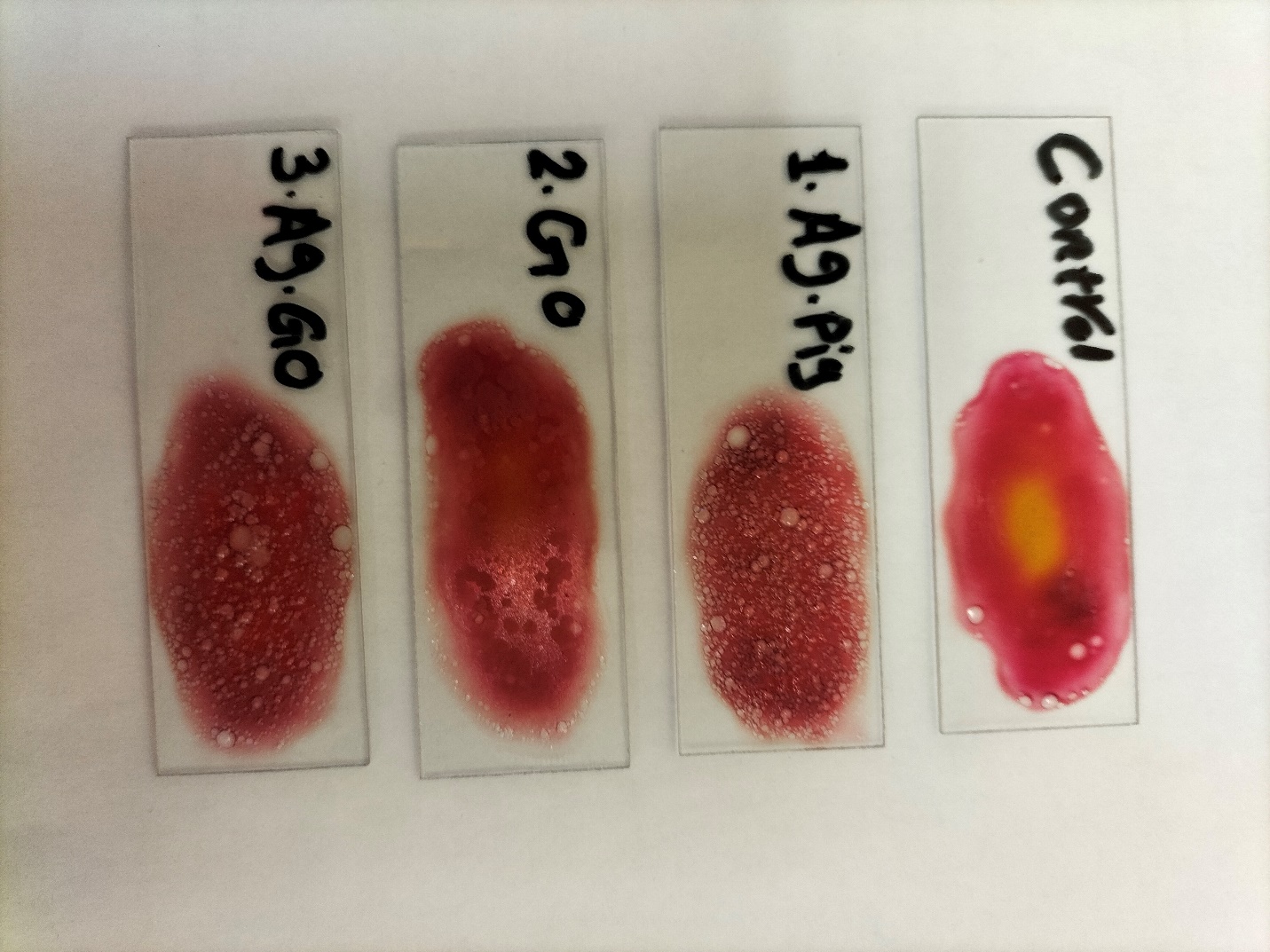

Supplement: Supplementary file 1 — Supplementary Material 1 [file 41598_2026_42211_MOESM1_ESM.docx]

## Graphical abstract

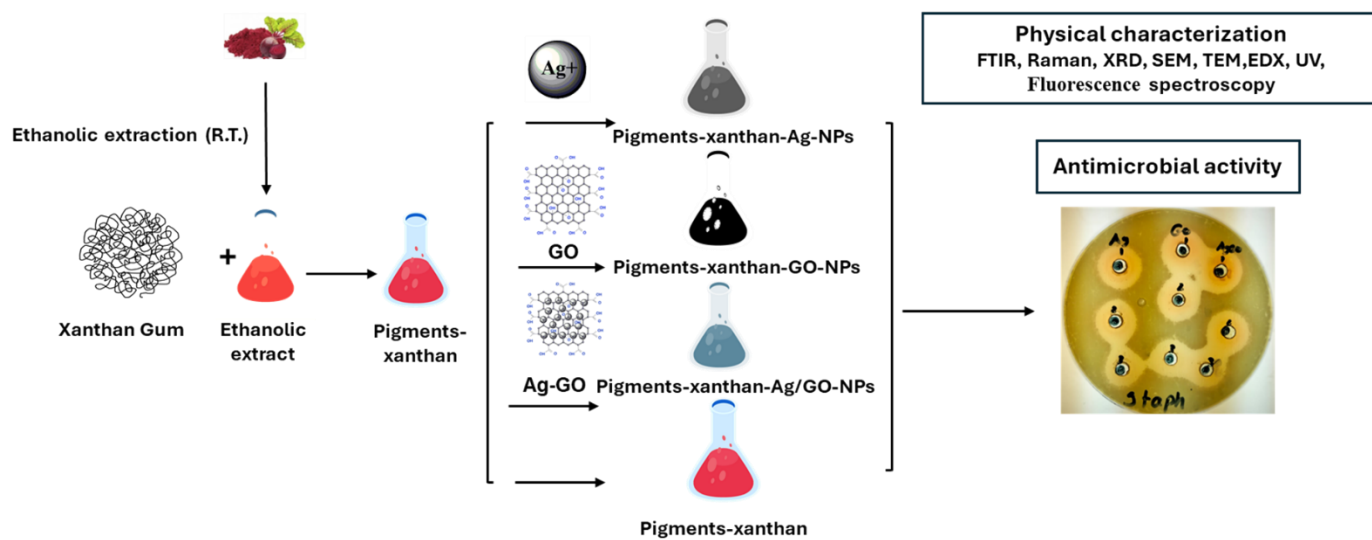

Supplement: Supplementary file 2 — Supplementary Material 2 [file 41598_2026_42211_MOESM2_ESM.pdf]
